# Close contacts involving germanium and tin in crystal structures: experimental evidence of tetrel bonds

**DOI:** 10.1007/s00894-017-3573-8

**Published:** 2018-01-08

**Authors:** Patrick Scilabra, Vijith Kumar, Maurizio Ursini, Giuseppe Resnati

**Affiliations:** NFMLab—D.C.M.I.C. “Giulio Natta”, Politecnico di Milano, Via L. Mancinelli 7, 20131 Milan, Italy

**Keywords:** Tetrel bond, Crystal engineering, σ-Hole interactions, Supramolecular interactions

## Abstract

Modeling indicates the presence of a region of low electronic density (a “σ-hole”) on group 14 elements, and this offers an explanation for the ability of these elements to act as electrophilic sites and to form attractive interactions with nucleophiles. While many papers have described theoretical investigations of interactions involving carbon and silicon, such investigations of the heavier group 14 elements are relatively scarce. The purpose of this review is to rectify, to some extent, the current lack of experimental data on interactions formed by germanium and tin with nucleophiles. A survey of crystal structures in the Cambridge Structural Database is reported. This survey reveals that close contacts between Ge or Sn and lone-pair-possessing atoms are quite common, they can be either intra- or intermolecular contacts, and they are usually oriented along the extension of the covalent bond formed by the tetrel with the most electron-withdrawing substituent. Several examples are discussed in which germanium and tin atoms bear four carbon residues or in which halogen, oxygen, sulfur, or nitrogen substituents replace one, two, or three of those carbon residues. These close contacts are assumed to be the result of attractive interactions between the involved atoms and afford experimental evidence of the ability of germanium and tin to act as electrophilic sites, namely tetrel bond (TB) donors. This ability can govern the conformations and the packing of organic derivatives in the solid state. TBs can therefore be considered a promising and robust tool for crystal engineering.

**Graphical abstract:**
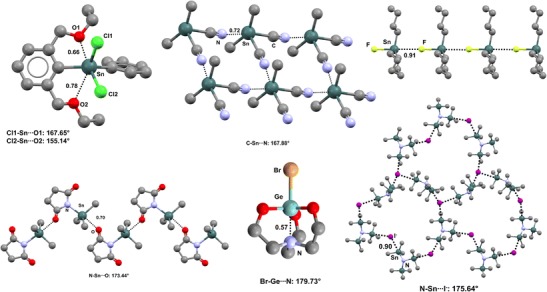
Intra- and intermolecular tetrel bonds involving organogermanium and -tin derivatives in crystalline solids

Intra- and intermolecular tetrel bonds involving organogermanium and -tin derivatives in crystalline solids

## Introduction

A comprehensive knowledge of the various interactions (i.e., weak bonds) that a molecule can participate in is a fundamental prerequisite for controlling and designing the conformation and the packing that the molecule adopts in a crystal. Interatomic distances that are slightly less than the sum of the van der Waals radii of the atoms involved (hereafter termed “close contacts”) are usually (but not always) the result of attractive interactions between the involved atoms. Observing the systematic occurrence of close contacts in crystalline solids can thus provide great insights into the attractive interactions that atoms and molecular moieties can participate in. Close contacts play a crucial role in the properties of matter, especially condensed phases, and knowledge and control of these contacts enables the functional properties of materials—synthetic and natural—to be designed and optimized [1–3].

Hydrogen bonds (HBs) are by far the most frequently occurring and widely studied type of interaction [4, 5]; other weak interactions that have traditionally received attention include π–π [6], cation–π [7], anion–π [8], and aurophilic [9] bonds. σ-Hole interactions [10–12] represent a relatively recent entry into the canon of weak bonds [13–15], but following the seminal papers of P. Politzer et al. [16, 17], these interactions rapidly became popular targets for studies in this field [15, 18–20]. A covalently bonded atom characteristically has a region of low electron density, known as the “σ-hole,” which is usually located along the extension of the covalent bond but on the opposite side of the atom to the bond. The electrostatic potential in this region is frequently positive and σ-hole bonding is the result of an attractive interaction between this positive region (an electrophilic site, the donor site in the interaction) and a negative site (a nucleophilic site, the acceptor site in the interaction, e.g., a lone-pair-possessing atom or an anion). In general, the largest number of σ-holes that an atom can have (which may drive the formation of attractive interactions) is equal to the number of the covalent bonds it is involved in. The more electron-withdrawing the group(s) covalently bound to a given atom is/are, the more extended and more positive the σ-hole(s) opposite to the bond(s) is/are [21], and the stronger and shorter the resulting σ-hole interaction(s) is/are. A distinctive feature of σ-hole interactions is their directionality, a consequence of the rather localized nature of the region(s) of positive electrostatic potential. In an R–A···B interaction, where A is the atom with the positive σ-hole potential and B is the nucleophile, the angle R–A···B is generally between 155° and 180°.

Experimental evidence and theoretical calculations consistently show that most of the elements in groups 14–18 of the periodic table form σ-hole bonds. A growing consensus is emerging among chemists that these interactions should be named according to the group of the periodic table that the electrophilic atom belongs to [22, 23]. Halogen bonds (XBs) [10, 24], namely interactions where an atom of a group 17 element is the electrophilic site, represent the best known subset of σ-hole interactions. Chalcogen bonds (CBs) have been studied in silico [25, 26] and in the solid [27], liquid [28], and gas [29] phases. Pnictogen bonds (PBs) have received much attention in studies performed in silico [30] and in the solid [31]; and the aerogen bond (AB) is the most recently discovered subset of σ-hole interactions [32].

The tetrel bond (TB), namely an interaction in which a group 14 element is the electrophile, has received a great deal of attention, probably due to the scale of its influence in chemistry, e.g., its possible role in S_N_2 reactions and hydrophobic interactions [13, 33]. The first convincing evidence of the ability of carbon to attractively interact with lone-pair-possessing atoms was reported more than forty years ago. In 1975, Johnson et al. calculated that the arrangement of the water–carbon dioxide dimer in which there is close C···O contact is more stable than the arrangement in which there is close H···O contact [34]. In 1984, Klemperer et al. confirmed, via microwave spectral analysis, that the equilibrium geometry of the adduct features a tetrel bond, i.e., that the tetrel-bonded O_2_C···OH_2_ geometry is preferred to the hydrogen-bonded HO–H···O=CO geometry [35]. During the 1980s, tetrel bonding was shown to be more important than hydrogen bonding for driving the formation of other lowest-energy complexes formed by carbon dioxide, for instance those with HBr [36] and HCN [37]. Most papers on the ability of tetrels to function as electrophiles describe theoretical investigations of interactions involving carbon [38] and silicon [39–41], whereas investigations of the heavier group 14 elements are far less frequent [42]. Experimental studies of TBs are quite limited [29, 43–45] and, to the best of our knowledge, they have never focused on interactions involving germanium or tin. We therefore decided to analyze structures in the Cambridge Structural Database (CSD) in order to assess whether organic derivatives of these two elements in crystalline solids show the presence of TBs. We looked for systems in which germanium and tin form close contacts with nucleophilic sites. Since directionality is a key characteristic of σ-hole interactions, particular attention was paid in this survey to the geometrical features of the observed close contacts, and a linear close contact was considered to be a TB.

In this paper, we discuss a selected number of crystalline structures of organic derivatives of germanium and tin in which these elements form TBs, i.e., close linear contacts with lone-pair-possessing heteroatoms. Structurally simple and poorly functionalized molecular systems are preferentially analyzed, as the Ge/Sn···nucleophile interactions that occur in these systems are more likely to be a straightforward product of the features of the two sites involved (contributions from other parts of the molecule(s) are likely to be insignificant). Wider coverage of organic Ge and Sn derivatives that present TBs in the solid is given in the works cited in this review. The interaction distances are analyzed based on the normalized contact (Nc), defined as the ratio between the experimentally observed separation of the interacting atoms and the sum of their respective van der Waals radii [46].^1^ The use of Nc values allows linear comparisons between contacts involving different atoms. While the number of CSD structures in which Ge/Sn···nucleophile interactions are present is not large enough to enable definitive and detailed generalizations to be made, the CSD survey reported here shows that the formation of attractive interactions between organic Ge and Sn sites and a donor of electron density can become a determinant of structure in crystalline solids. Intra- and intermolecular TBs are observed, and they can affect the preferred conformation of a molecule and/or the network of intermolecular interactions in the crystal packing. Importantly, the cases collected here provide convincing experimental evidence that TBs tend to be more linear than PBs [31].

## Oxygen atoms as TB acceptors

**Fig. 1:**
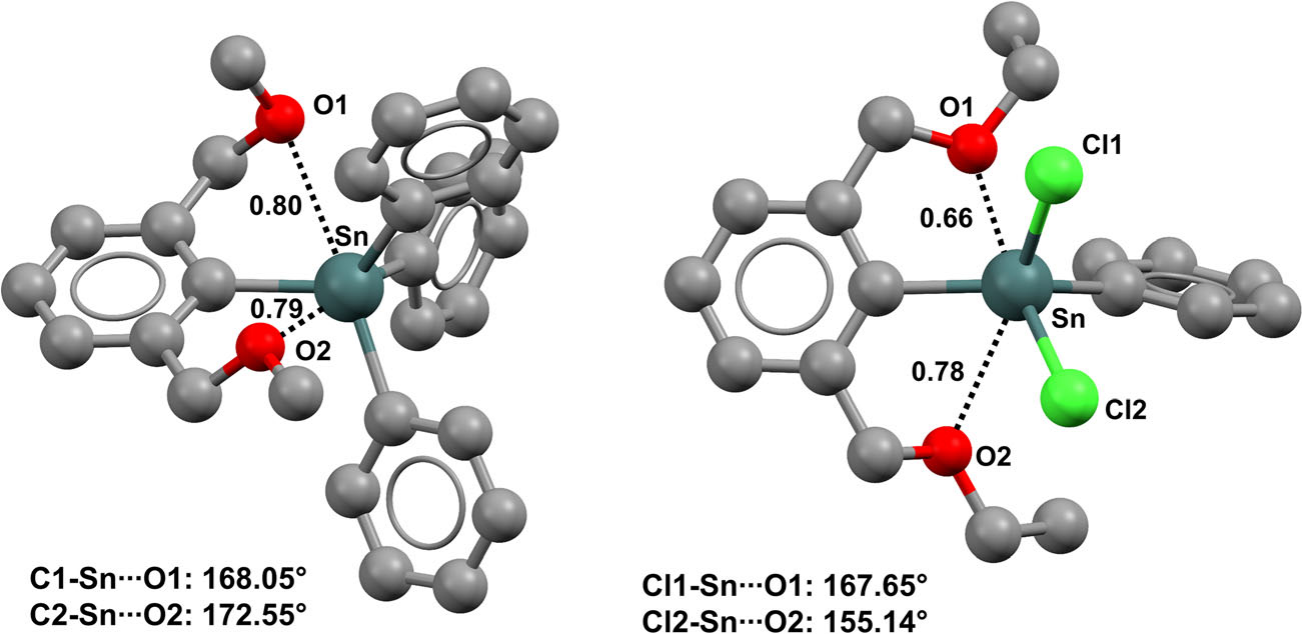
Ball and stick representations (Mercury 3.9) of (2,6-bis(methoxymethyl)phenyl)triphenyltin (MUBVOU,* left*) and (2,6-bis(ethoxymethyl)phenyl)dichlorophenyltin (LIVHOO,* right*). TBs are depicted as* black dotted lines*; hydrogens have been omitted for clarity. Nc values are shown close to the respective interactions. Color code:* gray* carbon,* green* chlorine,* red* oxygen,* dark teal* tin

The conformation adopted by (2,6-bis(methoxymethyl)phenyl)triphenyltin (refcode MUBVOU) in the crystal (Fig. 1, left) seems to be determined by two intramolecular Sn···O TBs [48]. One interaction distance is slightly shorter than the other, with the two Nc values being 0.76 and 0.78. Shorter σ-hole interactions usually tend to be more linear; consistent with this characteristic, the two C–Sn···O angles in the TBs mentioned above are 168.05° and 172.55°, respectively. As discussed above, another common feature of σ-hole interactions is that the more electron-withdrawing the residue covalently bonded to the σ-hole donor site, the more positive the σ-hole, and the closer and stronger the interactions with incoming nucleophiles. Interestingly, in an analog of the compound discussed above wherein two of the phenyl rings are replaced with chlorine atoms, the two intramolecular TBs are much shorter; i.e., in (2,6-bis(ethoxymethyl)phenyl)dichlorophenyltin (refcode LIVHOO), the Nc values for the Sn···O TBs are 0.66 and 0.78 (Fig. 1, right) [49].

**Fig. 2:**
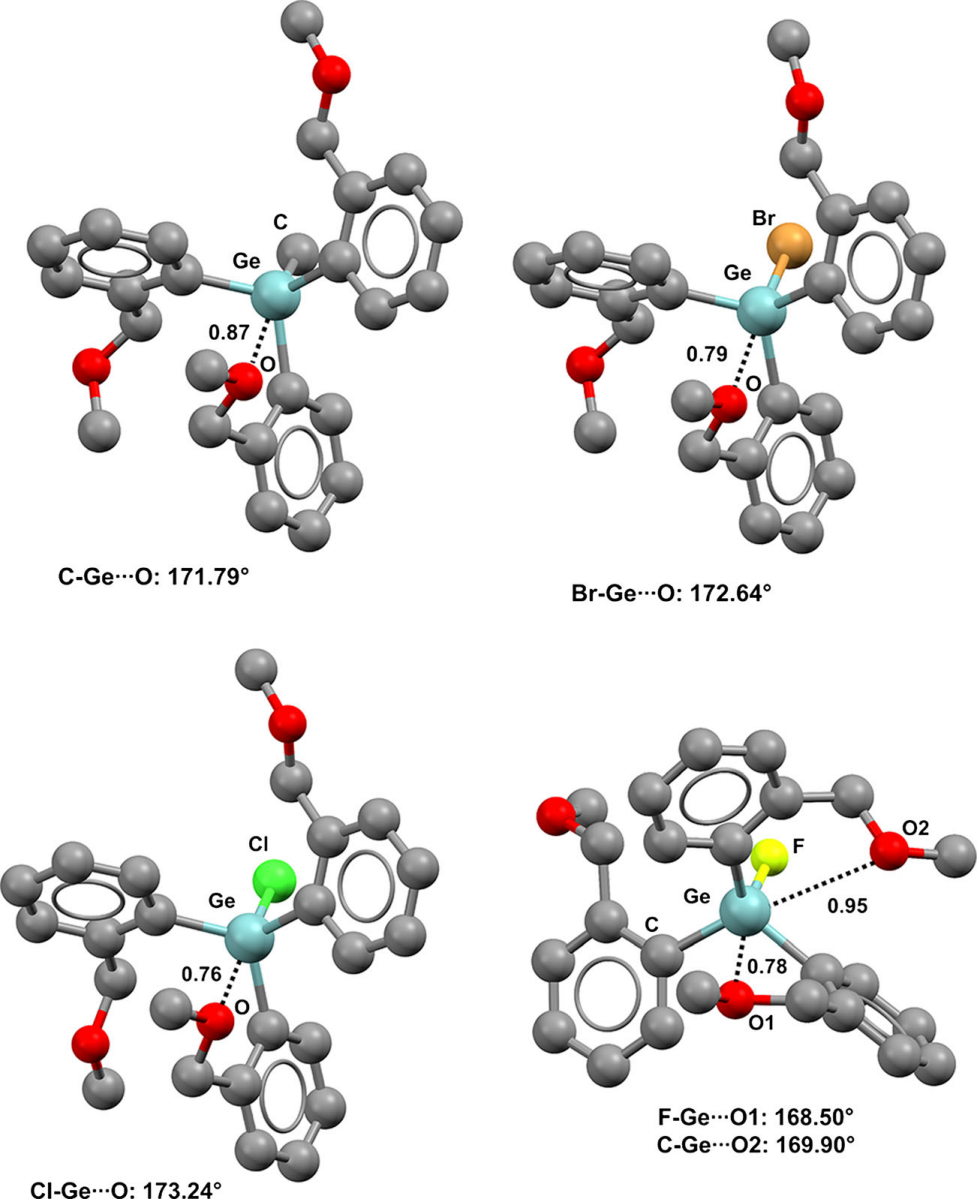
Ball and stick representations (Mercury 3.9) of methyl-tris((2-methoxymethyl)phenyl)germane (IMUTEP,* top left*), bromo-tris((2-methoxymethyl)phenyl)germane (IMUTAL,* top right*), chloro-tris((2-methoxymethyl)phenyl)germane (IMUSUE,* bottom left*), and fluoro-tris((2-methoxymethyl)phenyl)germane (IMUSOY,* bottom right*). TBs are depicted as* black dotted lines*; hydrogens have been omitted for clarity. Nc values are shown close to the respective interactions. Color code:* gray* carbon,* brown* bromine,* green* chlorine,* yellowish green* fluorine,* red* oxygen,* light teal* germanium

It is extensively documented that the propensity of a halogen atom to form XBs increases with its molecular weight [10], and that the heavier halogens usually form stronger and shorter XBs than the lighter ones, with both of these behaviors being independent of the XB acceptor. Similar trends are observed when elements of groups 16 and 15 form CBs and PBs, respectively. In all cases, this is probably due to the fact that within a group of the periodic table, the polarizability increases with the molecular weight of the element, and high polarizability favors an anisotropic distribution of the electron density around the atom and thus the strength of σ-hole interactions. It is no surprise [50] that methyl-tris((2-methoxymethyl)phenyl)germane (refcode IMUTEP) shows only one C–Ge···O contact, and that the corresponding Nc value (0.87) is greater than the Nc values of the structurally similar tin derivatives MUBVOU and LIVHOO [51] (Fig. 2, top left).

**Fig. 3:**
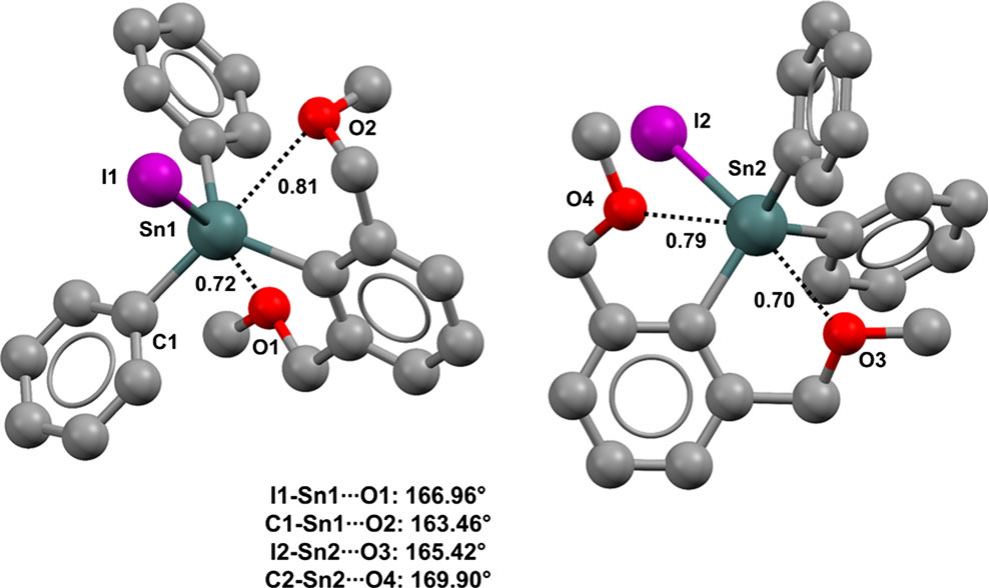
Ball and stick representation (Mercury 3.9) of the two molecules of the unit cell of iodo(2,6-bis(methoxymethyl)phenyl)diphenyltin (RAKBOV). TBs are depicted as* black dotted lines*; hydrogen atoms have been omitted for clarity. Nc values are shown close to the respective interactions. Color code:* gray* carbon,* red* oxygen,* purple* iodine,* dark teal* tin

Bromine is more electronegative than carbon, and the Br–Ge···O TB in bromo-tris((2-methoxymethyl)phenyl)germane (refcode IMUTAL) is shorter (Nc = 0.79) than the C–Ge···O in IMUTEP (Fig. 2, top right) [51]; chlorine is more electronegative than bromine, and the Cl–Ge···O TB in chloro-tris((2-methoxymethyl)phenyl)germane (refcode IMUSUE) is even shorter (Nc = 0.76) (Fig. 2, bottom left) than the Br–Ge···O TB. Also, in these three structures, the linearity of the TB is correlated with its length (the C–Ge···O, Br–Ge···O, and Cl–Ge···O angles are 171.79°, 172.64°, and 173.24°, respectively). In fluoro-tris((2-methoxymethyl)phenyl)germane (refcode IMUSOY), a fluorine is substituted for the methyl of IMUTEP and the depletion of electron density at germanium becomes large enough that two TBs are present (Fig. 2, bottom right). Consistent with the relative electronegativities of fluorine and carbon, the F–Ge···O interaction is closer and more directional than the C–Ge···O interaction (the Nc values for the Ge···O separations are 0.78 and 0.95, respectively). Also, the presence of a tin-bonded iodine atom can promote the formation of close contacts (Fig. 3). Two independent molecules are present in the unit cell of crystalline iodo(2,6-bis(methoxymethyl)phenyl)diphenyltin (refcode RAKBOV), and in both of them the conformation is locked in by two intramolecular TBs: an I–Sn···O and a C–Sn···O TB. The distances of the former interactions are shorter and those interactions are more directional than the latter ones (the Nc values are 0.70 and 0.72 for I–Sn···O and 0.79 and 0.81 for C–Sn···O; the mean I–Sn···O angle is 166.19° and the mean C–Sn···O angle is 166.68°).

**Fig. 4:**
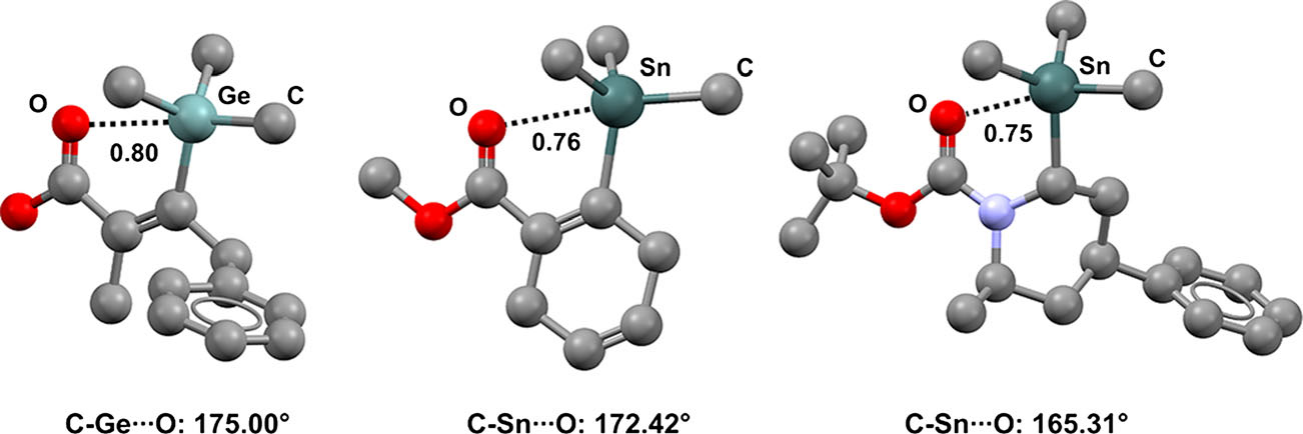
Ball and stick representations (Mercury 3.9) of one of the two independent molecules in the unit cells of (*Z*)-2-methyl-4-phenyl-3-(trimethylgermanyl)but-2-enoic acid (QIBDOV,* left*), (2-carbomethoxy-1,4-cyclohexadien-1-yl)trimethyltin (KASYOS,* middle*), and *trans*-*N*-*t*-butyloxycarbonyl-2-methyl-6-(trimethylstannyl)-4-phenyl)piperidine (EABFES,* right*) derivatives. TBs are depicted as* black dotted lines*; hydrogen atoms have been omitted for clarity. Nc values are shown close to the respective interactions. Color code:* gray* carbon,* red* oxygen,* light blue* nitrogen,* light teal* germanium,* dark teal* tin

Carbonyl oxygen atoms can act as effective TB acceptors. In (*Z*)-2-methyl-4-phenyl-3-(trimethylgermanyl)but-2-enoic acid (refcode QIBDOV) [52], a short C–Ge···O contact is present in both conformations adopted by the compound in the crystals (Fig. 4, left) (Nc for Ge···O is 0.80; the C–Ge···O angles are 174.17° and 175.00°), and a shorter TB occurs in a trimethylstannylcarbomethoxy derivative (refcode KASYOS) [53], where a similar tin-based tecton is present (Nc for C–Sn···O is 0.76) (Fig. 4, middle). Similar TBs are given by the carbonyl oxygens of carbamates (e.g., *N*-*t*-butyloxycarbonyl-2-methyl-6-trimethylstannyl-4-phenyl-piperidine, refcode EABFES; Nc = 0.75 and the C–Sn···O angle is 165.31°; Fig. 4, right) [54, 55] and several other carbonyl derivatives, e.g., amides [56], aldehydes [57, 58], and ketones [59].

**Fig. 5:**
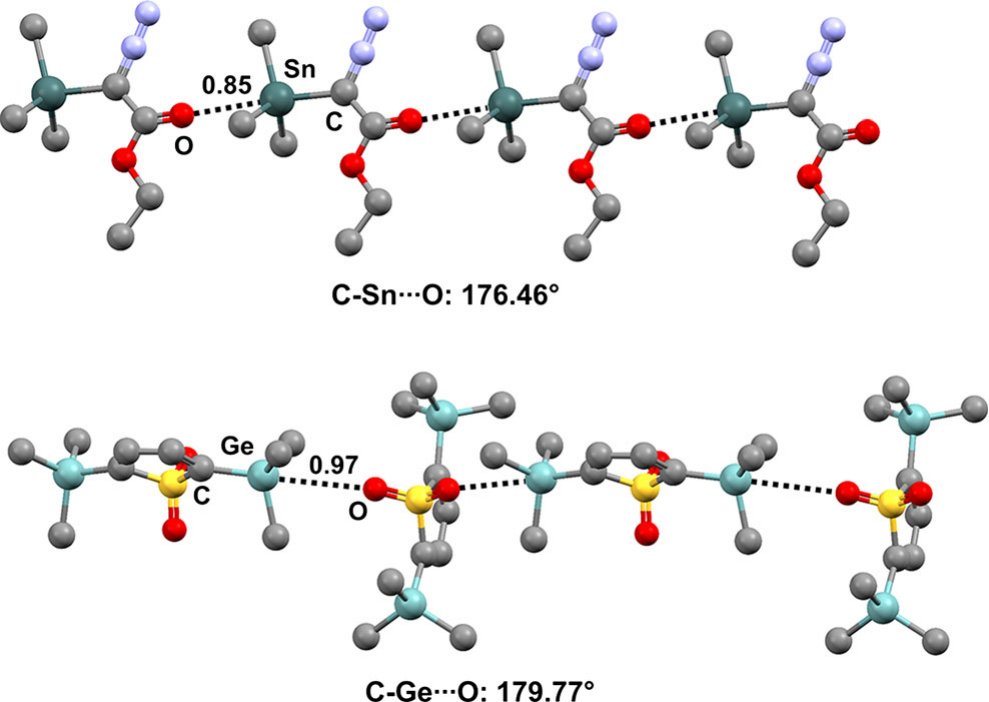
Ball and stick representations (Mercury 3.9) of 1D chains generated by ethyl trimethyltin diazoacetate (SIWRAR,* top*) and 2,5-bis(trimethylgermyl)thiophene-1,1-dioxide (QAHXIG,* bottom*). TBs are depicted as* black dotted lines*; hydrogen atoms have been omitted for clarity. Nc values are shown close to the respective interactions. Color code:* gray* carbon,* red* oxygen,* light blue* nitrogen,* yellow* sulfur,* light teal* germanium,* dark teal* tin

The CSD contains both intra- and intermolecular TBs that have a carbonyl oxygen acting as the TB acceptor and facilitate the generation of discrete adducts [60] or infinite chains (one-dimensional networks, 1D nets). In ethyl trimethyltin diazoacetate (refcode SIWRAR) [61], the diazoacetate residue is expected to form a σ-hole on tin that is more positive than the σ-holes formed by the methyl groups. Consistent with this expectation, a tetrel-bonded infinite chain is present in the crystal of the compound (Fig. 5, top), wherein the carbonyl oxygen approaches the tin atom along the extension of the N_2_C–Sn covalent bond (the Sn···O separation is 312.5 pm, which corresponds to an Nc value of 0.85; the C–Sn···O angle is 176.46°). Similarly, the most positive σ-hole on germanium in 2,5-bis(trimethylgermyl)thiophene-1,1-dioxide (refcode QAHXIG) [62] is expected to occur opposite to the O_2_SC–Ge covalent bond, and an infinite chain (Fig. 5, bottom) is formed in which the sulfonyl oxygens approach germanium atoms along the extension of each O_2_SC–Ge covalent bond, leading to a particularly linear geometry (the Ge···O separation corresponds to an Nc value of 0.97, and the C–Ge···O angle is 179.77°).

**Fig. 6:**
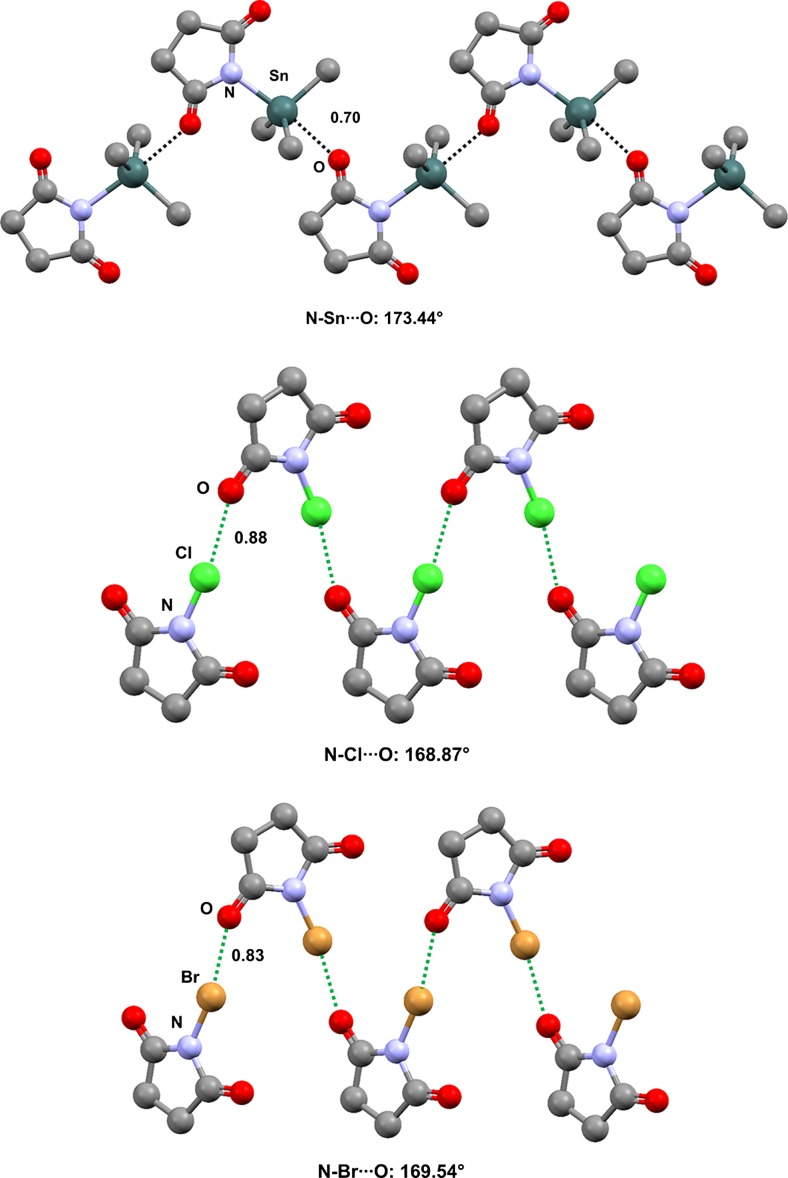
Ball and stick representations (Mercury 3.9) of the 1D networks formed by *N*-triethylstannylsuccinimide (FUSZIC) due to N–Sn···O TBs (*top*), by* N*-chlorosuccinimide (CSUCIM) due to N–Cl···O XBs (*middle*), and by *N*-bromosuccinimide (NBSUCA) due to N–Br···O XBs (*bottom*). The three methyl groups of the ethyl residues of *N*-triethylstannylsuccinimide and hydrogen atoms have been deleted for the sake of simplicity. TBs and XBs are depicted as* black dotted lines* and* green dotted lines*, respectively. Color code:* gray* carbon,* red* oxygen,* purple* iodine,* brown* bromine,* dark teal* tin

*N*-triethylstannylsuccinimide (refcode FUSZIC) [63] is a self-complementary module that forms tetrel-bonded infinite chains (one-dimensional networks, 1D nets) (Fig. 6, top). Consistent with the expected involvement of an *sp*^2^ lone pair of the carbonyl oxygen as the nucleophilic site that interacts with Sn along the extension of the N–Sn covalent bond, the Sn···O=C angle is 138.28° and the tin atom is approximately in the plane of the succinimide (the distance between the mean square plane through the seven heavy atoms of the succinimide moiety and the tetrel-bonded tin atom is 219 pm). The halogen-bonded infinite chains formed by *N*-chloro- and* N*-bromosuccinimide (refcodes CSUCIM01 and NBSUCA, respectively) [64] are also reported in Fig. 6 (middle and bottom) in order to highlight the analogous supramolecular features of TB and XB.

**Fig. 7:**
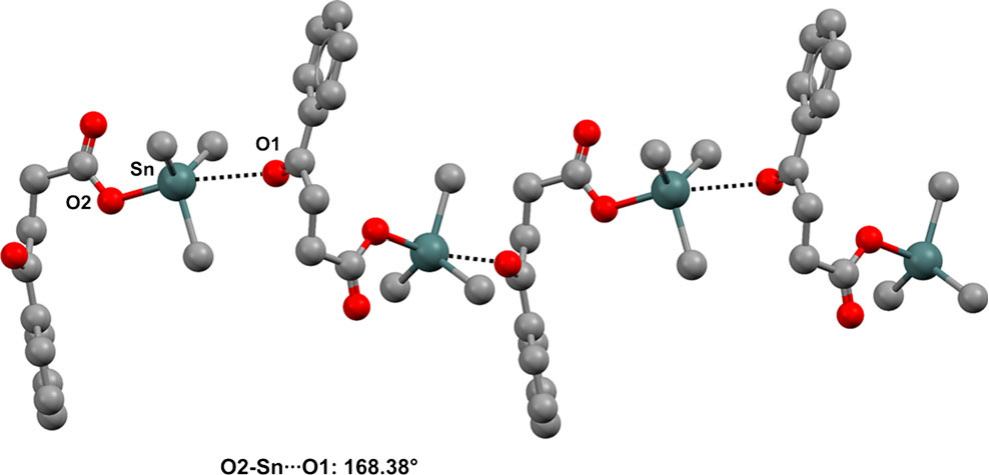
Ball and stick representation (Mercury 3.9) of the 1D network in which the ketone oxygen of* O*-tricyclohexyltin-4-oxo-4-phenylbutanoate (APAZIB) functions as the TB acceptor site. Hydrogen atoms and five of the cyclohexyl carbons have been deleted for the sake of simplicity. Color code:* gray* carbon,* red* oxygen,* dark teal* tin

**Fig. 8:**
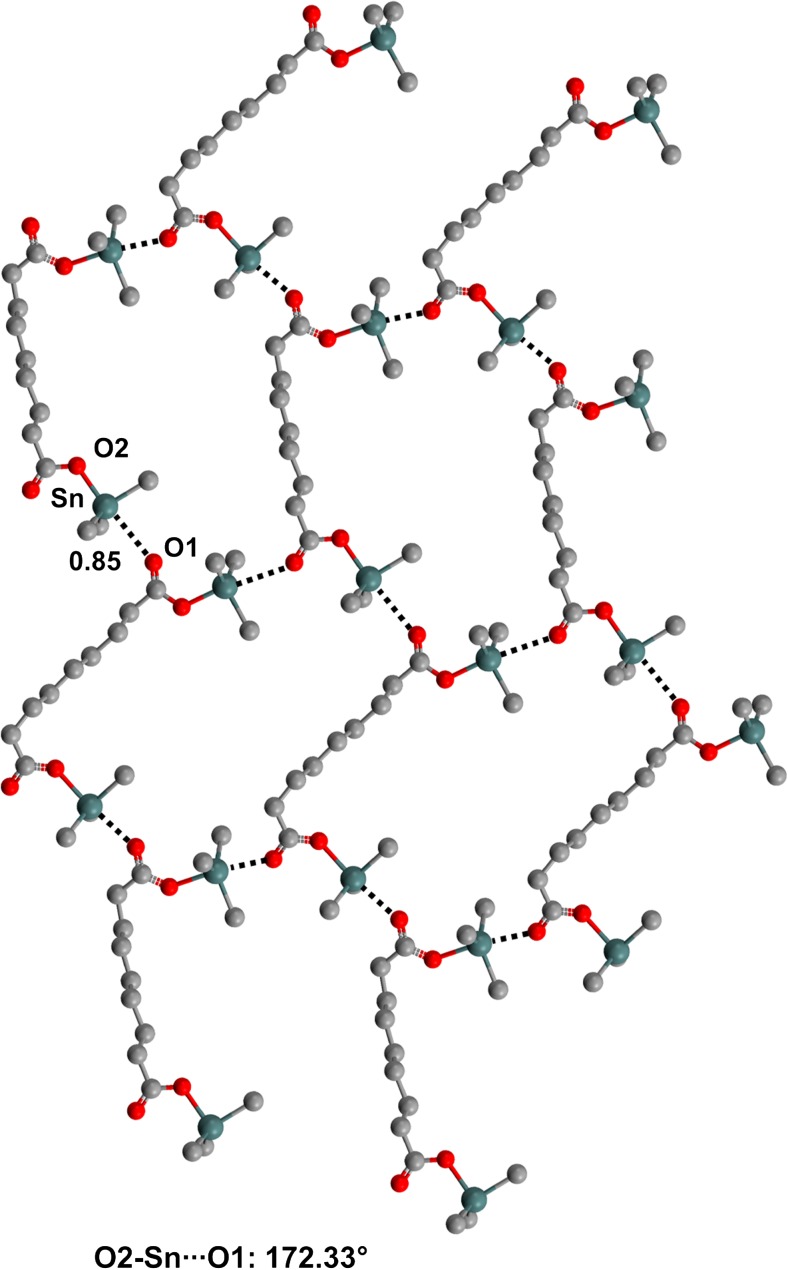
Ball and stick representation (Mercury 3.9) of the two-dimensional network formed by bis(tricyclohexyltin)nonanoate (CUXSOF). Five atoms of the cyclohexyl residues bound to tin have been deleted for the sake of simplicity. Color code:* gray* carbon,* red* oxygen,* dark teal* tin

**Fig. 9:**
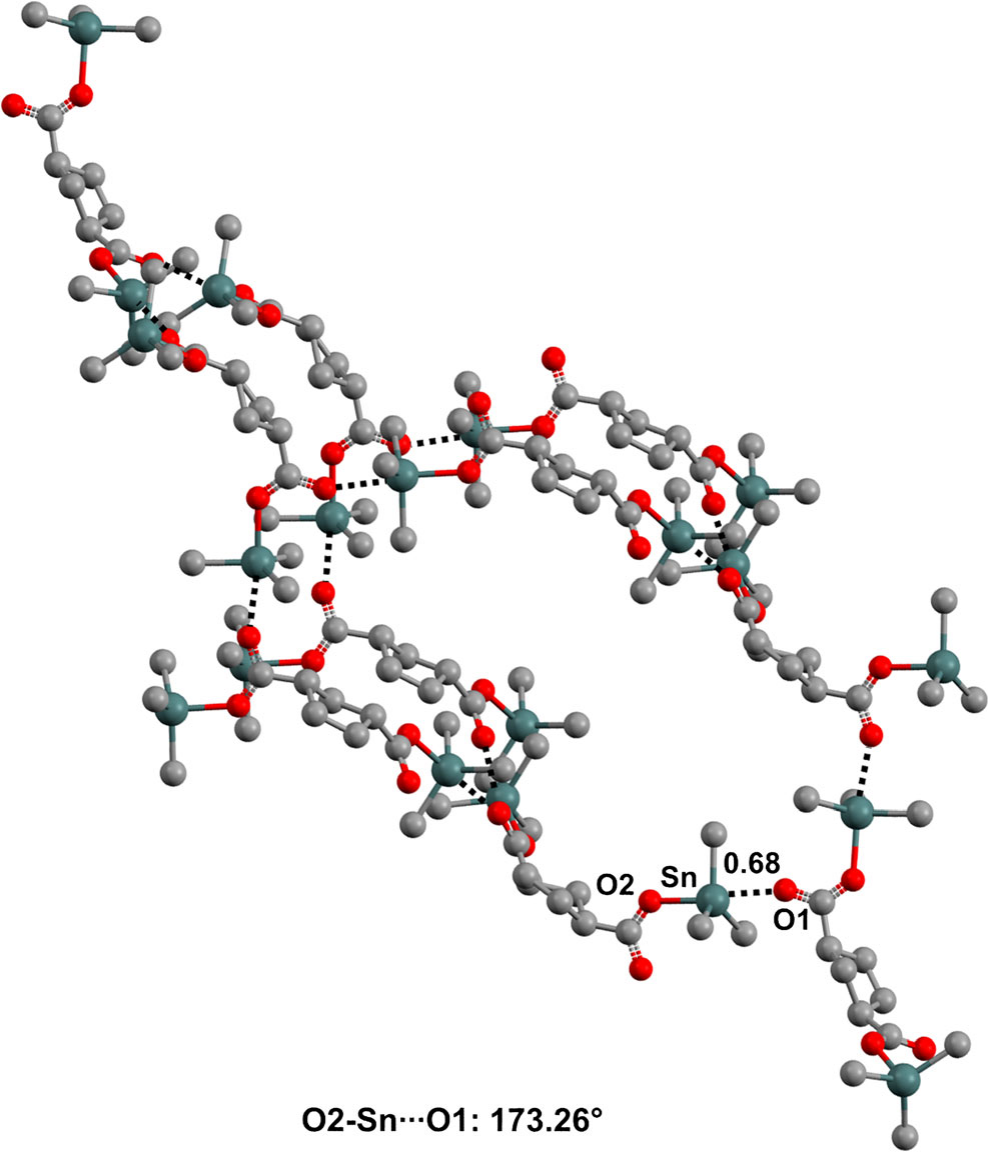
Ball and stick representation (Mercury 3.9) of the three-dimensional network with adamantanoid topology formed by bis(tri-*n*-butyltin)-1,2,2-trimethylcyclopentane-1,3-dicarboxylate (DIYFIB). Three atoms of the butyl residues bound to tin and the methyl pendants on the cyclopentyl rings have been deleted for the sake of simplicity. Color code:* gray* carbon,* red* oxygen,* dark teal* tin

In several structures in the CSD, the tin atom of a trialkylalkanoyltin moiety found in R_3_Sn–OC(O)R′ derivatives shows the presence of a TB with a carbonyl oxygen located opposite to the covalent Sn–O bond, and one-dimensional [65], two-dimensional [66], or three-dimensional [67] networks are formed depending on the overall structure of the compound (Figs. 7, 8, and 9).

**Fig. 10:**
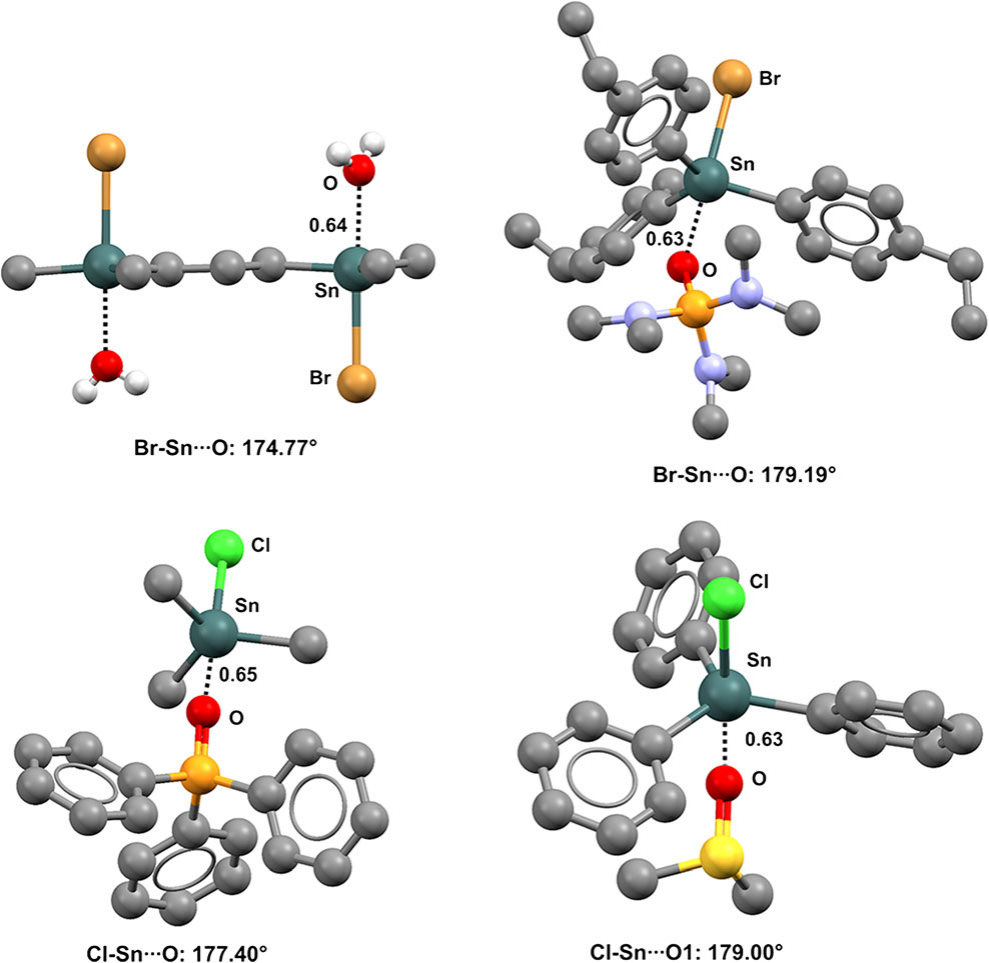
Ball and stick representations (Mercury 3.9) of the trimer formed by 1,3-bis(bromodimethylstannyl)propane and water (XINROB,* top left*), of the dimer formed by bromo-tris(*p*-ethylphenyl)tin and hexamethylphosphoramide (HEVQIJ,* top right*), of the dimer formed by chlorotrimethyltin and triphenylphosphine oxide (HIGRUK01,* bottom left*), and of the dimer formed by chlorotriphenyltin and dimethyl sulfoxide (RUGYOI,* bottom right*). Hydrogen atoms and the 2,2′-bipyridine in XINROB have been deleted for the sake of simplicity. TBs are depicted as* black dotted lines*. Color code:* gray* carbon,* red* oxygen,* blue* nitrogen,* orange* phosphorus,* green* chlorine,* brown* bromine,* yellow* sulfur,* dark teal* tin

Various other oxygen functionalities can act as donors of electron density to organotin and germanium derivatives, e.g., water [68–70], sulfoxides and sulfones [71–74], as well as phosphine oxides, hexamethylphosphortriamide, and their analogs [75–81] (Fig. 10).

## Nitrogen atoms as TB acceptors

**Fig. 11:**
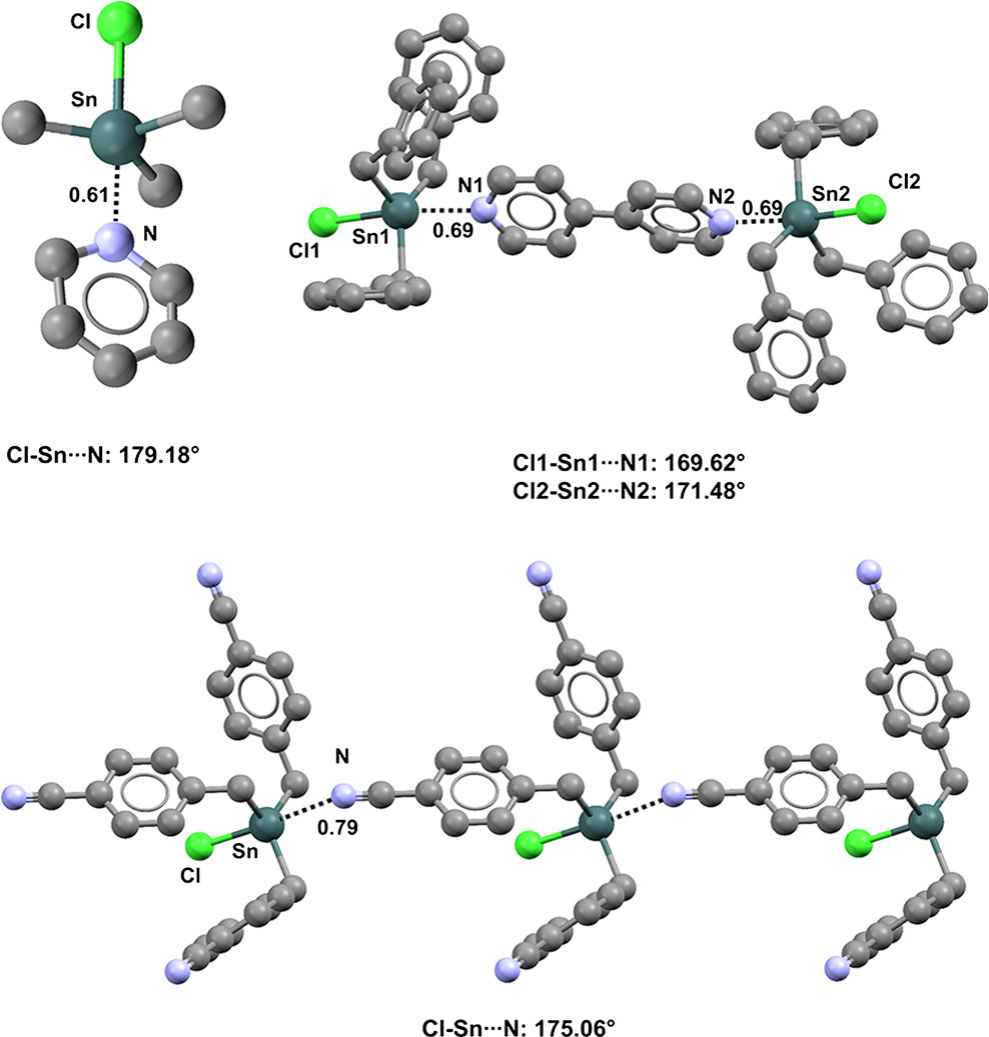
Ball and stick representations (Mercury 3.9) of the dimer formed by chloro(trimethyl)tin and pyridine (CMEPSN,* top left*), of the trimer formed by chloro(tribenzyl)tin and 4,4′-bipyridyl (FEJFUW,* top right*), and of the 1D chain formed by chlorotris(4-cyanobenzyl)tin (BIBQIN,* bottom*). Hydrogen atoms have been deleted for the sake of simplicity. TBs are depicted as* black dotted lines*. Color code:* gray* carbon,* blue* nitrogen,* green* chlorine,* dark teal* tin

The CSD contains several structures in which the nitrogen atom of amine, pyridine, and cyano moieties forms a close contact with a tin or germanium atom (Fig. 11), thus showing that—similar to oxygen atoms—nitrogen atoms can act as TB acceptors, and this can be the case whether there is *sp*^3^, *sp*^2^, or *sp* hybridization.

**Fig. 12:**
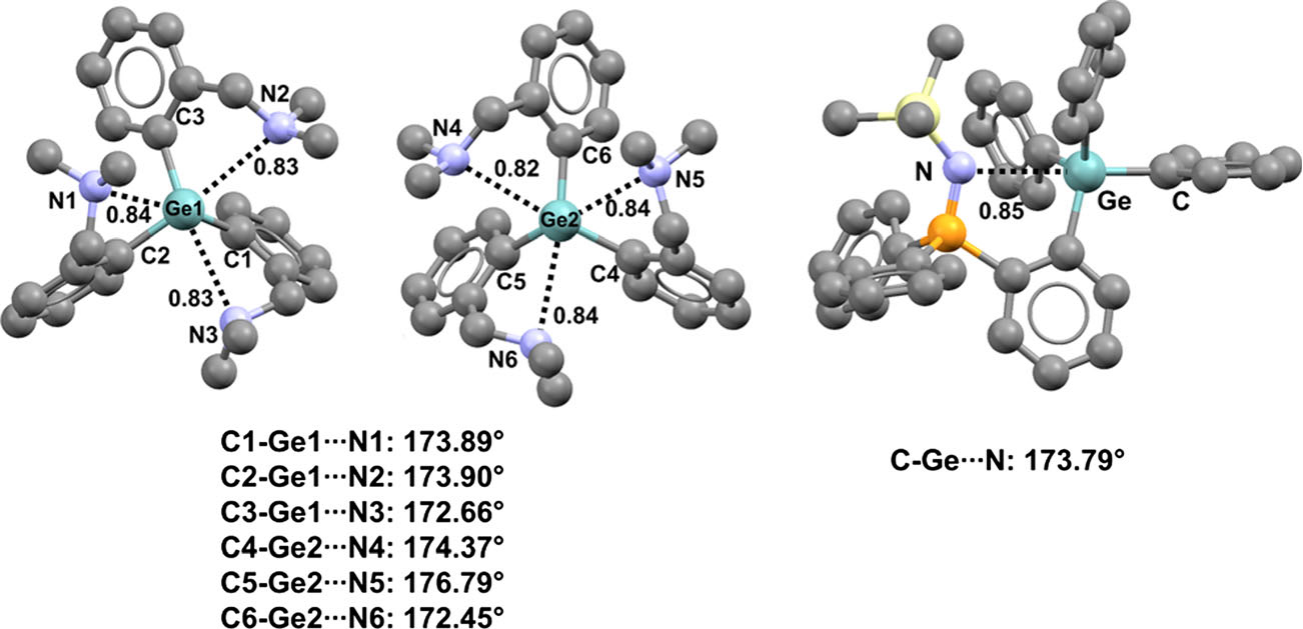
Ball and stick representations (Mercury 3.9) of tris(2-((dimethylamino)methyl)phenyl)germane (GAGYIW,* left*) and 1-(trimethylsilylimino(diphenyl)phosphoranyl)-2-(triphenylgermyl)benzene (VIQXIC,* right*) derivatives. TBs are depicted as* black dotted lines*; hydrogen atoms have been omitted for clarity. Nc values are shown close to the respective interactions. Color code:* gray* carbon,* light blue* nitrogen,* yellow* sulfur,* pearl white* silicon,* orange* phosphorus,* light teal* germanium

The ability of nitrogen atoms of tertiary amines to form close contacts with organogermanium and -tin derivatives is particularly well documented. For instance, two symmetrically nonequivalent molecules are present in crystals of tris(2-((dimethylamino)methyl)phenyl)germane (refcode GAGYIW) [82], and the conformations of both molecules are influenced by three intramolecular C–Ge···N TBs (Fig. 12, left) (the Nc values of these TBs span the range 0.82–0.84, and the C–Ge···N angles vary between 172.45° and 176.79°). The C–N–C angles vary between 109.70° and 113.25°, indicating that nitrogen atoms of the tertiary amine moieties adopt a tetrahedral conformation and the lone pairs align with the extensions of the covalent C–Ge bonds, as expected for σ-hole interactions (the C–N···Ge angles span the range 82.34–120.39°).

Imine nitrogen atoms behave in a similar manner to amine nitrogens. A close linear C–Ge···N interaction affects the conformation adopted by 1-(trimethylsilylimino(diphenyl)phosphoranyl)-2-(triphenylgermyl)benzene (Nc for Ge···N is 0.85; the C–Ge···N angle is 173.79°) (refcode VIQXIC) [83] (Fig. 12, right). In the crystal of this compound, the P=N···Ge angle is 96.80°, and the germanium atom is approximately in the iminophosphoranyl plane (the distance between the tetrel-bonded germanium atom and the mean square plane through the phosphorus, nitrogen, and silicon atoms is 263 pm), suggesting that the lone pair at nitrogen aligns with the extension of the covalent C–Ge bond.

**Fig. 13:**
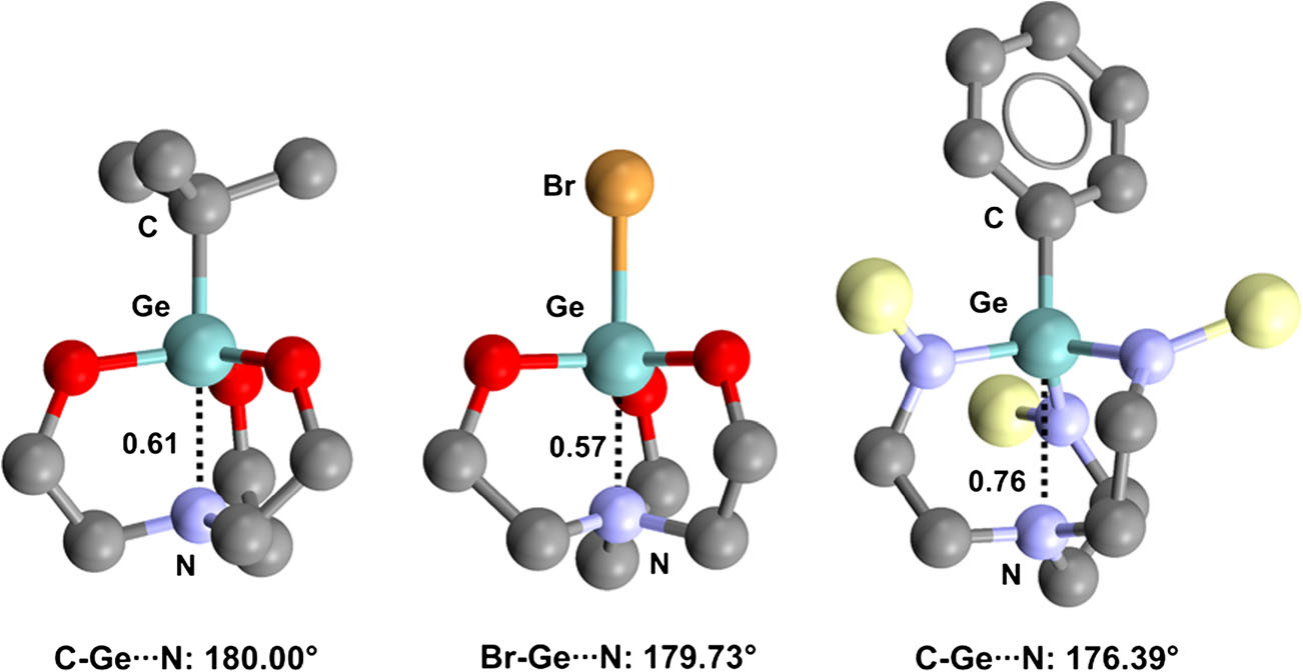
Ball and stick representations (Mercury 3.9) of 5-(*t*-butyl)germatrane (BUWBUQ,* left*), 5-bromogermatrane (BUWCUR,* middle*), and phenyl(tris(2-(trimethylsilylamido)ethyl)amine-*N*,*N*′,*N*″)germanium (XUSLOM,* right*). TBs are depicted as* black dotted lines*; hydrogen atoms and methyl substituents on the silyl moieties of XUSLOM have been omitted for clarity. Nc values are shown close to the respective interactions. Color code:* gray* carbon,* red* oxygen,* light blue* nitrogen,* bronze* bromine,* pearl white* silicon,* light teal* germanium

**Fig. 14:**
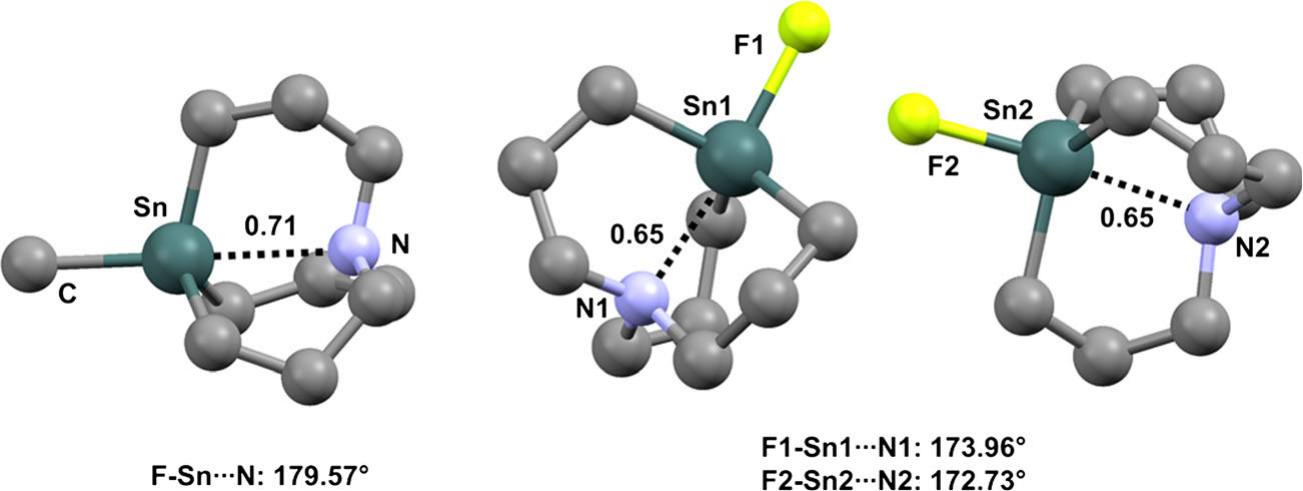
Ball and stick representations (Mercury 3.9) of 5-methyl-1-aza-5-stannabicyclo(3.3.3)undecane (FEWXOU,* left*) and 5-fluoro-1-aza-5-stannatricyclo(3.3.3)undecane (ZANKEE,* right*). TBs are depicted as* black dotted lines*; hydrogen atoms have been omitted for clarity. Nc values are shown close to the respective interactions. Color code:* gray* carbon,* light blue* nitrogen,* yellowish green* fluorine,* dark teal* tin

Close intramolecular Ge···N contacts affect the conformation of a family of 4,6,11-trioxa-1-aza-5-germabicyclo[3.3.3]undecanes (germatrane derivatives). In the solid, 5-(*t*-butyl)-germatrane (refcode BUWBUQ) [84] adopts an *endo* conformation (Fig. 13, left) where the C–Ge···N separation is as short as 223.6 pm (Nc = 0.61). 5-Bromogermatrane (refcode BUWCUR) [85] behaves similarly (Fig. 13, middle), and the Br–Ge···N separation is even shorter (208.4 pm, Nc = 0.57) than in BUWBUQ, consistent with the fact that bromine is more electronegative than carbon and the σ-hole opposite the Br–Ge covalent bond is probably more positive than that opposite the C–Ge bond. Analogous *endo* conformations and Ge···N distances that are much shorter than the sum of the van der Waals radii of the germanium and nitrogen atoms are observed in other germatrane derivatives [86–88] and related systems [89, 90] (Fig. 13, right). Similar behavior is encountered in the crystals of tin analogs. 5-Methyl-1-aza-5-stannabicyclo[3.3.3]undecane (refcode FEWXOU) [79] and its 5-fluoro [91], 5-chloro [92], 5-bromo [91], and 5-iodo [91] analogs (refcodes ZANKEE, DAYMUL, ZANKOO, ZANKUU, respectively) all show close Sn···N contacts (Fig. 14).

**Fig. 15:**
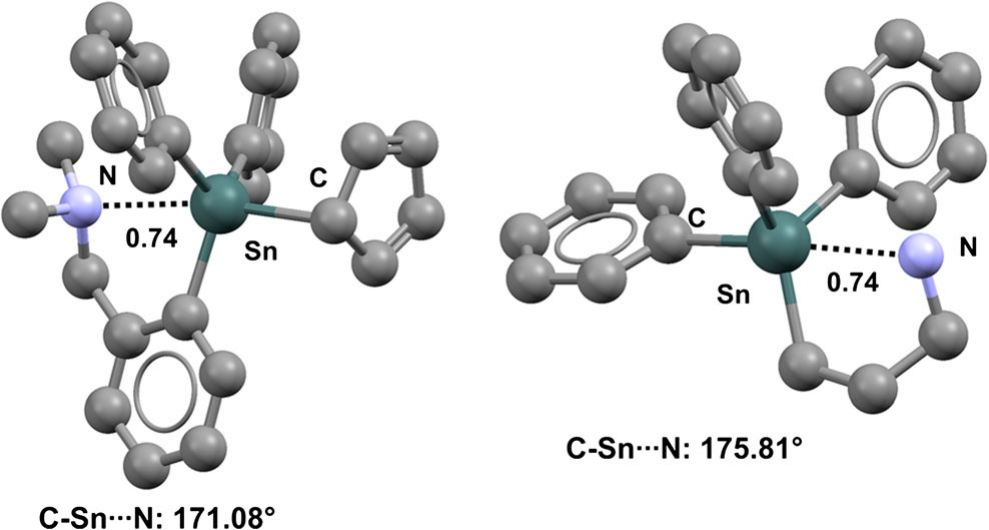
Ball and stick representations (Mercury 3.9) of (cyclopenta-2,4-dien-1-yl)-(2-(dimethylaminomethyl)phenyl)diphenyltin (*left*) and (3-aminopropyl)triphenyltin (IHOZAH,* right*). TBs are depicted as* black dotted lines*; hydrogen atoms have been omitted for clarity. Nc values are shown close to the respective interactions. Color code:* gray* carbon,* light blue* nitrogen,* dark teal* tin

As in organogermanium derivatives, the nitrogen atom of the 2-(dimethylaminomethyl)phenylstannyl moiety forms an intramolecular TB which affects the conformation of the respective compound in the solid. This is the case for (cyclopenta-2,4-dien-1-yl)-(2-(dimethylaminomethyl)phenyl)diphenyl tin (refcode IHOZAH) [93] (Fig. 15, left), where the intramolecular C–Sn···N distance corresponds to an Nc value of 0.74 and the C–Sn···N angle is 171.08°, congruent with an attractive interaction between the lone pair of the tertiary amine nitrogen and the σ-hole along the extension of the C–Sn covalent bond. Analogous Sn···N interactions are present in structurally related derivatives [94–96]. A five-membered and tetrel-bonded ring similar to that of IHOZAH is afforded by (3-aminopropyl)triphenyltin (refcode COKVUV) [97] (Fig. 15, right), which shows an Sn···N interaction where Nc is 0.74 and the C–Sn···N angle is 175.81°.

**Fig. 16:**
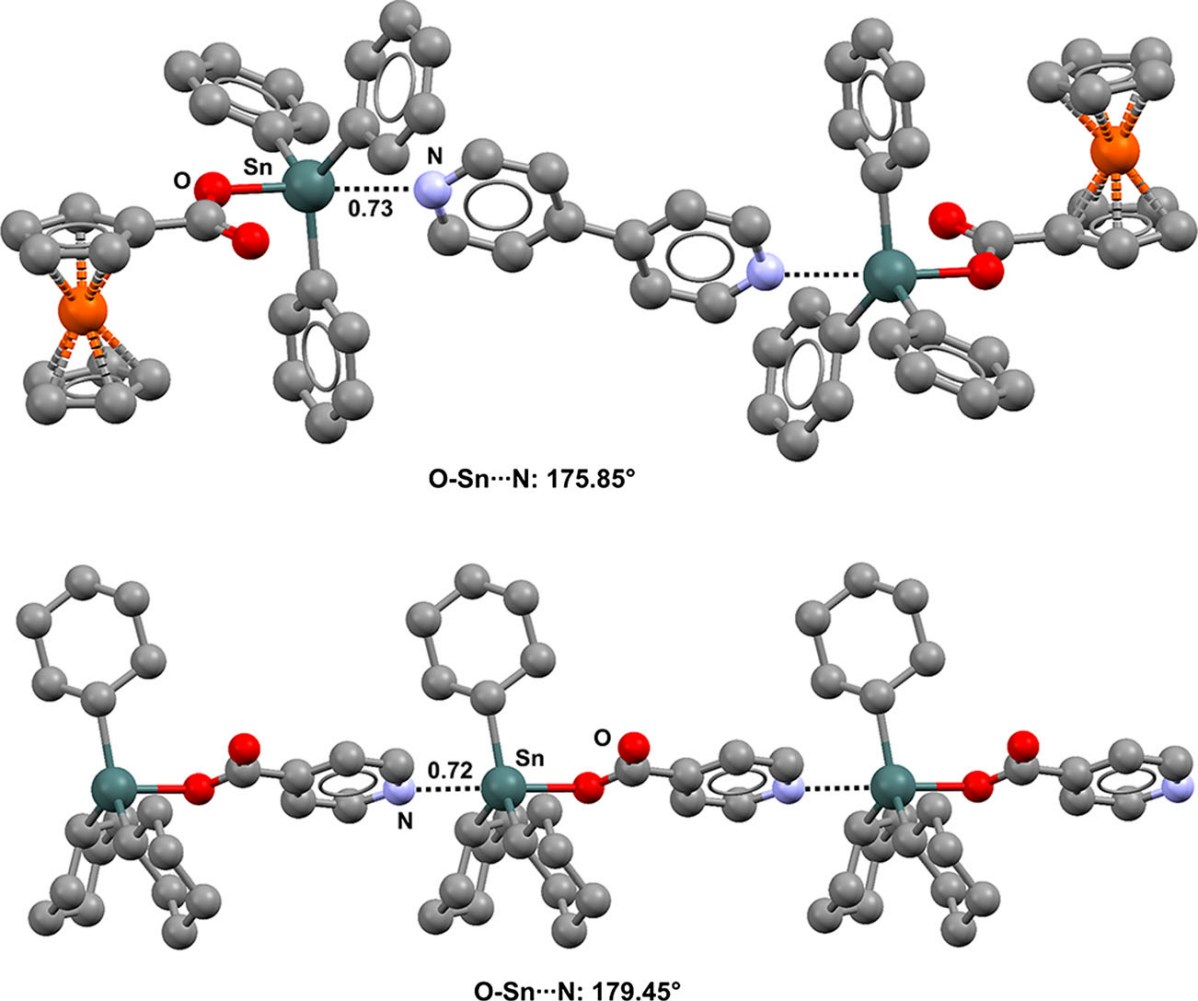
Ball and stick representations (Mercury 3.9) of the trimer formed by (ferrocene-1-carboxylato)triphenyltin and 4,4′-bipyridine (IVUVUR,* top*) and of the infinite chain formed by (pyridine-4-carboxylato)tricyclohexyltin (UZAVUN,* bottom*). TBs are depicted as* black dotted lines*; hydrogen atoms have been deleted for the sake of simplicity. Nc values are shown close to the respective interactions. Color code:* gray* carbon,* red* oxygen,* orange* iron,* light blue* nitrogen,* dark teal* tin

**Fig. 17:**
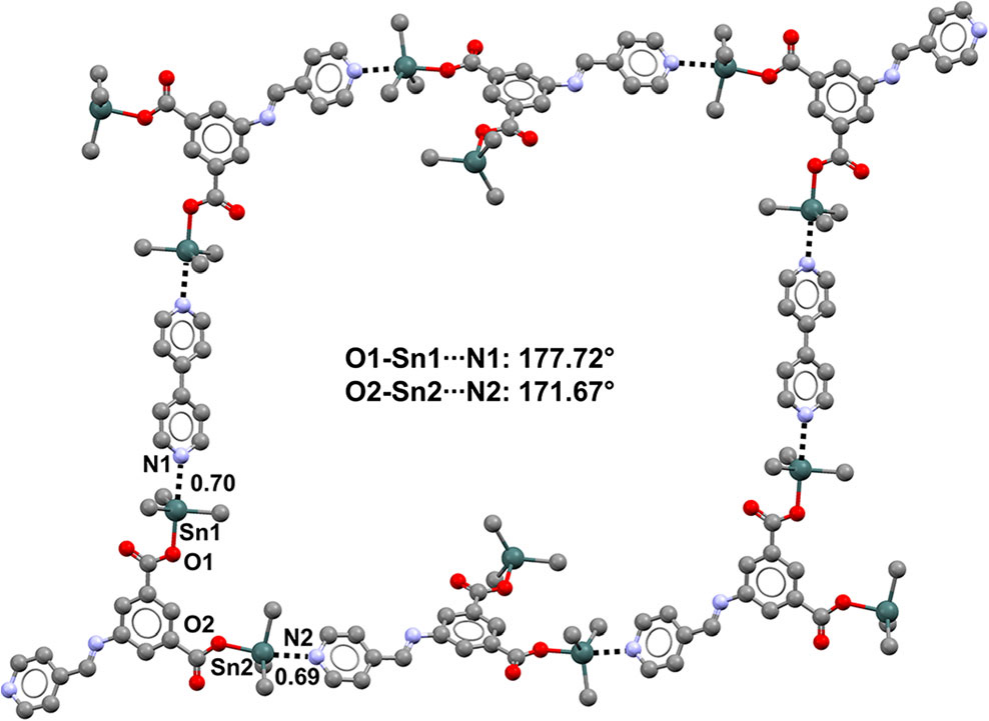
Ball and stick representation (Mercury 3.9) of the network generated by di(tri-*n*-butyl)stannyl-5-((pyridin-4-ylmethylene)amino)isophthalate with 4,4′-bipyridine (TISVEY). TBs are depicted as* black dotted lines*; three atoms of the butyl residues at tin and hydrogen atoms have been deleted for the sake of simplicity. Nc values are shown close to the respective interactions. Color code:* gray* carbon,* red* oxygen,* light blue* nitrogen,* dark teal* tin

The tin atom of R_3_Sn–OC(O)R′ derivatives is a good TB donor and frequently interacts with the oxygen atom of a carbonyl group (Figs. 7–9) or the nitrogen atom of a pyridine moiety. The intermolecular Sn···N interaction occurs opposite to the covalent Sn–O bond, and discrete trimers [98] (Fig. 16, top) or one-dimensional [99–102] (Fig. 16, bottom) or two-dimensional [103] networks (Fig. 17) are formed depending on the ability of the tin derivative to function as a mono-, bi-, or polydentate tecton.

**Fig. 18:**
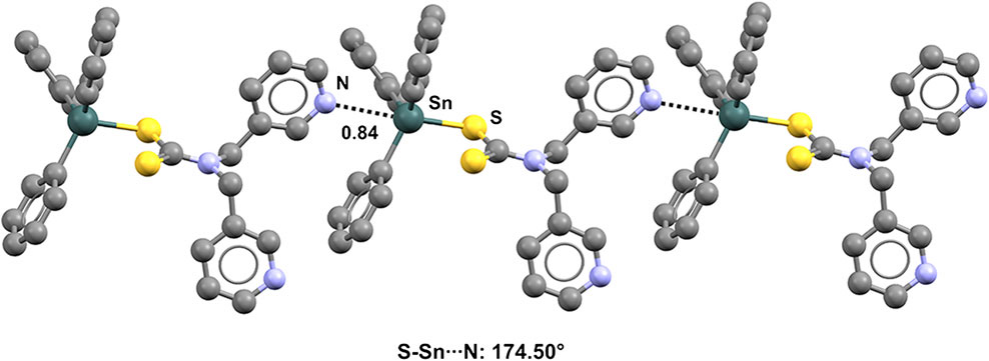
Ball and stick representation (Mercury 3.9) of the one-dimensional network formed by (bis(pyridin-3-ylmethyl)carbamodithioato)triphenyltin (UGEFIX). TBs are depicted as* black dotted lines*; hydrogen atoms have been deleted for the sake of simplicity. Nc values are shown close to the respective interactions. Color code:* gray* carbon,* yellow* sulfur,* light blue* nitrogen,* dark teal* tin

The nitrogen atom of pyridine derivatives forms close contacts with tin along the extensions of not only O–Sn bonds but also C–Sn, Cl–Sn, Br–Sn, I–Sn, and S–Sn bonds [99, 101, 104, 105]. In all cases, the geometric features of the adducts indicate that the nitrogen lone pair is oriented along the extension of one of the covalent bonds of tin. For instance, in the infinite chain formed by the dithiocarbamate reported in Fig. 18 (refcode UGEFIX), the S–Sn···N angle is 174.50°, the geometry around the nitrogen is strictly trigonal planar, and tin is nearly in the pyridine plane (the two C(*sp*^2^)N···Sn angles are 121.18° and 122.40°, and the distance of tin from the mean square plane through the pyridine ring is 85 pm).

**Fig. 19:**
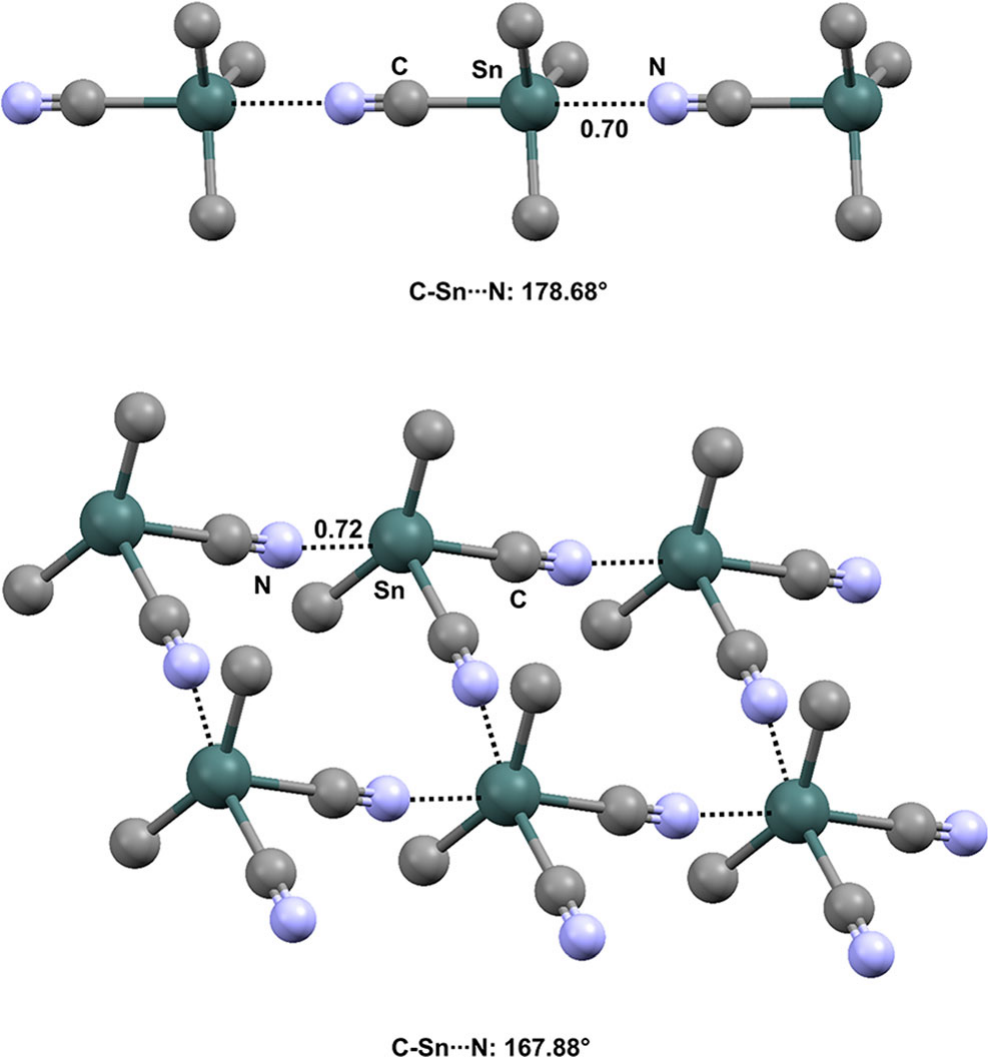
Ball and stick representations (Mercury 3.9) of the 1D infinite chain formed by trimethyltin cyanide (TIMSNC01,* top*) and the 2D network generated by dimethyltin dicyanide (DMCYSN,* bottom*). TBs are depicted as* black dotted lines*; hydrogen atoms have been deleted for the sake of simplicity. Nc values are shown close to the respective interactions. Color code:* gray* carbon,* light blue* nitrogen,* dark teal* tin

The cyano group seems to be able to act as an effective TB acceptor group via the lone pair at the nitrogen. Moreover, due to its strong electron-withdrawing ability, it is expected that when the cyano group is directly bound to a tin or germanium atom, the σ-hole opposite the covalent NC–Sn/Ge bond will be particularly positive. Indeed, trimethyltin cyanide (refcode TIMSNC01) and dimethyltin dicyanide (refcode DMCYSN) are both self-complementary modules that form infinite chains [106] and square 2D networks [107], respectively (Fig. 19), by pairing TB donor and TB acceptor sites. Dimethylgermanium dicyanide (refcode DMCYGE) shows somewhat similar behavior.

**Fig. 20:**
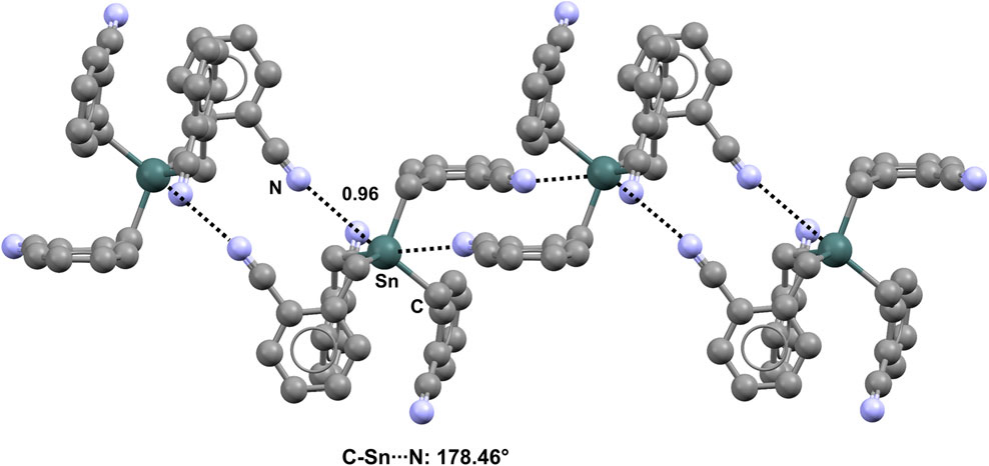
Ball and stick representation (Mercury 3.9) of the network formed by tetrakis(2-cyanobenzyl)tin (JIWROX). TBs are depicted as* black dotted lines*; hydrogen atoms have been deleted for the sake of simplicity. Nc values are shown close to the respective interactions. Color code:* gray* carbon,* light blue* nitrogen,* dark teal* tin

Tetrakis(2-cyanobenzyl)tin (refcode JIWROX) [108] (Fig. 20) functions as a self-complementary tecton, as the cyano group of one molecule aligns with the extension of one of the C–Sn covalent bonds of an adjacent molecule, ultimately forming infinitely long tetrel-bonded ribbons (Nc = 0.96; the C–Sn···N angle is 178.46°).

**Fig. 21:**
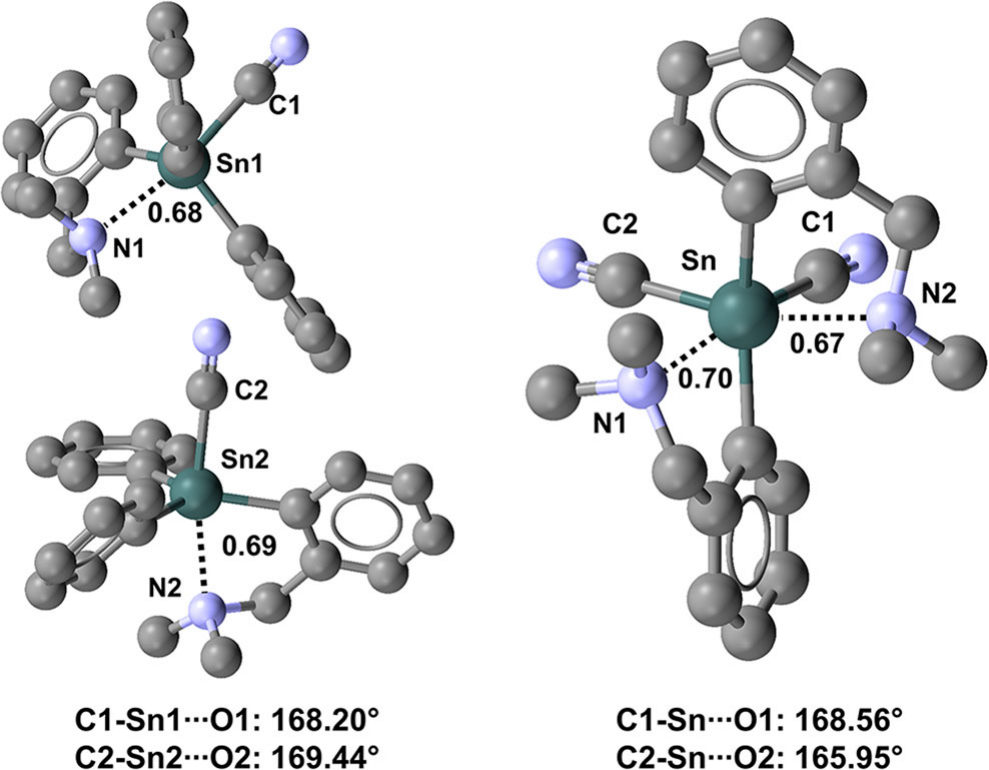
Ball and stick representations (Mercury 3.9) of the conformations adopted by cyano-2-(dimethylaminomethyl)phenyl)diphenyltin (WUVKOP,* left*) and bis(2-(dimethylaminomethyl)phenyl)dicyanotin (WUVLOQ,* right*). TBs are depicted as* black dotted lines*; hydrogen atoms have been deleted for the sake of simplicity. Nc values are shown close to the respective interactions. Color code:* gray* carbon,* light blue* nitrogen,* dark teal* tin

In 2-(dimethylaminomethyl)phenyl)cyanodiphenyltin and bis(2-(dimethylaminomethyl)phenyl)dicyanotin (refcodes WUVKOP and WUVLOQ, respectively) [109], one and two NC–Sn···N close contacts are present, respectively, and the amine nitrogen acts as the TB acceptor site in all cases (Fig. 21). This may suggest that a N(*sp*^3^) atom is a better TB acceptor than a N(*sp*) atom. The same ability to act as a donor of electron density is observed in XB formation.

## Halogen atoms as tetrel bond acceptors

Structures in the CSD reveal that the four halogens F, Cl, Br, and I can all form close contacts with tetravalent germanium and tin atoms in organic derivatives. These interactions can be rationalized as TBs due to the fact that the halogen atom is located approximately along the extension of one of the covalent bonds formed by the germanium or tin. The bond with the most electron-withdrawing group is preferentially involved in the formation of these close contacts.

**Fig. 22:**
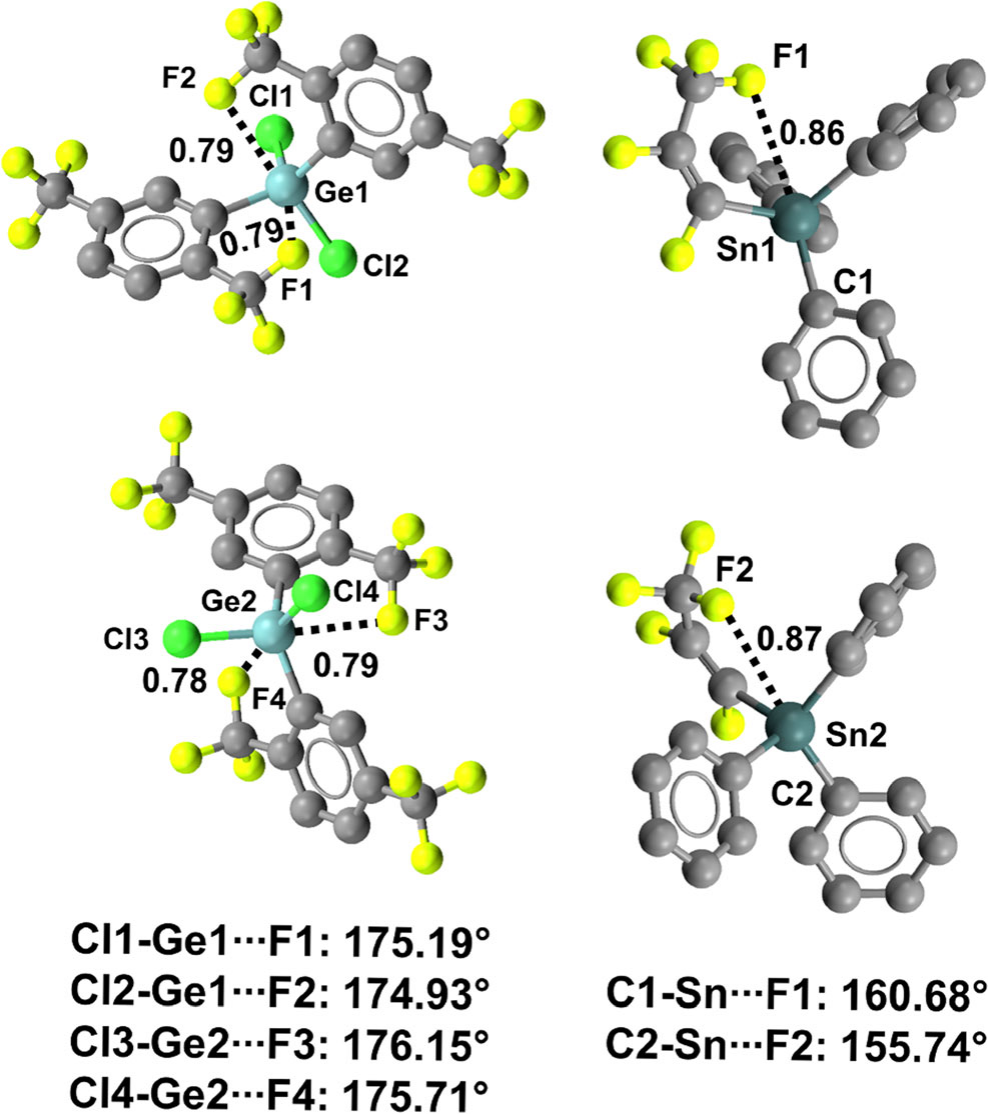
Ball and stick representations (Mercury 3.9) of the conformations adopted by bis(2,5-bis(trifluoromethyl)phenyl)(dichloro)germane (ZAVCUW,* left*) and (1,2,3,3,3-pentafluoroprop-1-en-1-yl)triphenyltin (ADUKOB,* right*). TBs are depicted as* black dotted lines*; hydrogen atoms have been deleted for the sake of simplicity. Nc values are shown close to the respective interactions. Color code:* gray* carbon,* yellowish green* fluorine,* light teal* germanium,* dark teal* tin

For instance, crystals of bis(2,5-bis(trifluoromethyl)phenyl)(dichloro)germane (refcode ZAVCUW) have two symmetrically nonequivalent molecules in the unit cell [110]. Both of these molecules show two fairly short and linear TBs oriented along the extensions of the Cl–Ge bonds (Nc values span the range 0.78–0.79; the C–Ge···F angles are between 176.15° and 174.93°) (Fig. 22, left). Analogously, an intramolecular C–Ge···F close contact locks in the conformation of (1,2,3,3,3-pentafluoroprop-1-en-1-yl)triphenylgermanium (refcode ADUKUH) [111] in the solid and allows for the formation of a tetrel-bonded five-membered ring (the Nc value of Ge···F is 0.86; the C–Ge···F angle is 166.80°). The tin analog of ADUKUH (refcode ADUKOB) behaves similarly, as an intramolecular C–Sn···F TB is present in both of the independent molecules present in the unit cell of the crystal, leading to a tetrel-bonded ring (Fig. 22, right).

**Fig. 23:**
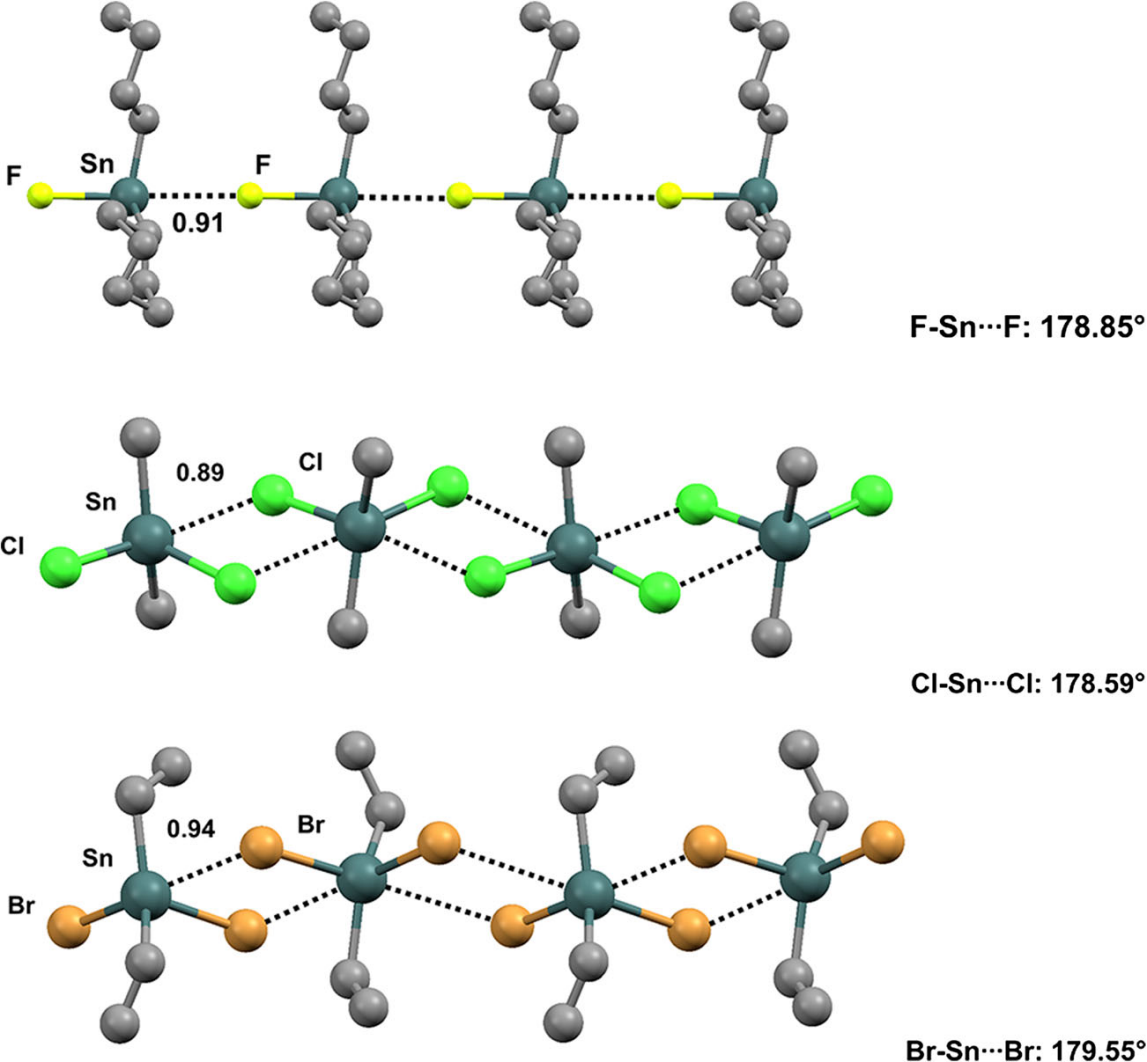
Ball and stick representations (Mercury 3.9) of 1D chains generated by fluorotricyclohexyltin (BAJWOY,* top*), dichlorodimethyltin (DMSNCL,* middle*), and dibromodiethyltin (DESNBR,* bottom*). TBs are depicted as* black dotted lines*; hydrogen atoms have been omitted for clarity. Nc values are shown close to the respective interactions. Color code:* gray* carbon,* brown* bromine,* green* chlorine,* yellowish green* fluorine,* dark teal* tin

Interestingly, tricyclohexyltin fluoride (refcode BAJWOY) [112] is a self-complementary module, as the fluorine atom of one molecule forms a short and remarkably linear TB along the extension of the covalent F–Sn bond of an adjacent molecule, and infinitely long chains are generated (Fig. 23, top) (the Nc value for Sn···F is 0.91; the F–Sn···F angle is 178.85°). Similar behavior is shown by several other organotin derivatives bearing one, two, or three halogen atoms at the heavy tetrel [113–116] (Fig. 23).

**Fig. 24:**
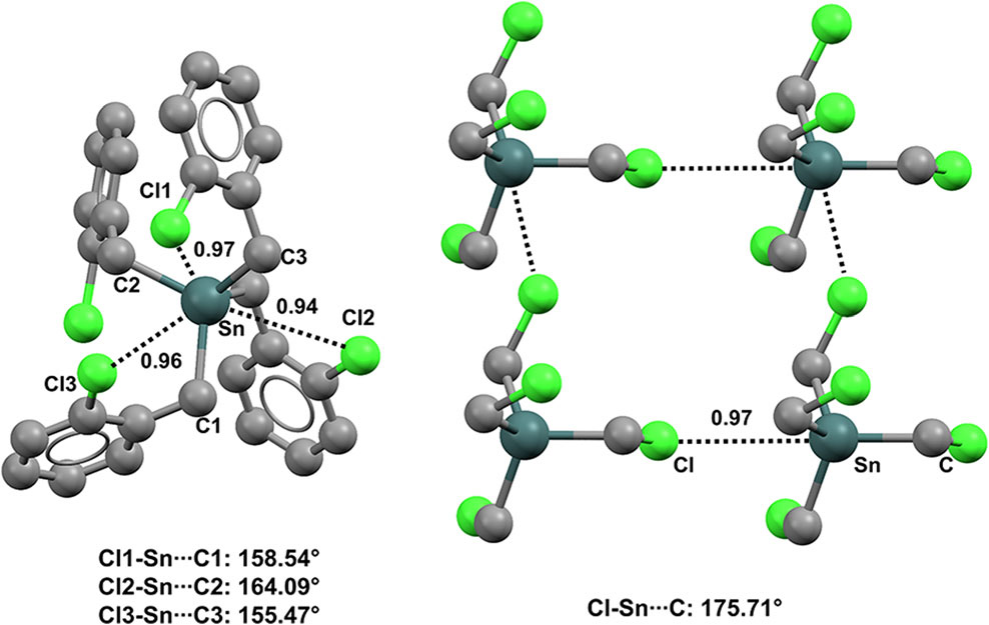
Ball and stick representations (Mercury 3.9) of the conformation adopted by tetrakis(2-chlorobenzyl)tin (CEWGEQ01,* left*) and the network generated by tetrakis(chloromethyl)tin (UGATEB,* right*). TBs are depicted as* black dotted lines*; hydrogen atoms have been omitted for clarity. Nc values are shown close to the respective interactions. Color code:* gray* carbon,* green* chlorine,* dark teal* tin

Crystals of tetrakis(2-chlorobenzyl)tin (refcode CEWGEQ) [117] provide a nice example of intramolecular C–Sn···Cl interactions, as three such contacts (Nc values range from 0.94 to 0.97) lock in the molecular conformation (Fig. 24, left). Interestingly, tetrakis(2-methoxybenzyl)tin (refcode HEVFOD) [118] and tetrakis(2-fluorobenzyl)tin (refcode VULSOM) [119] present four intramolecular C–Sn···O and C–Sn···F TBs in their respective crystals. Tetrakis(chloromethyl)tin (refcode UGATEB) [120] also provides a good example of intermolecular C–Sn···Cl contacts. The molecule is a self-complementary bidentate TB donor (at tin) and acceptor (at chlorine) (Fig. 24, right), and a tetrel-bonded (4,4) network is formed wherein UGATEB sits at the nodes.

**Fig. 25:**
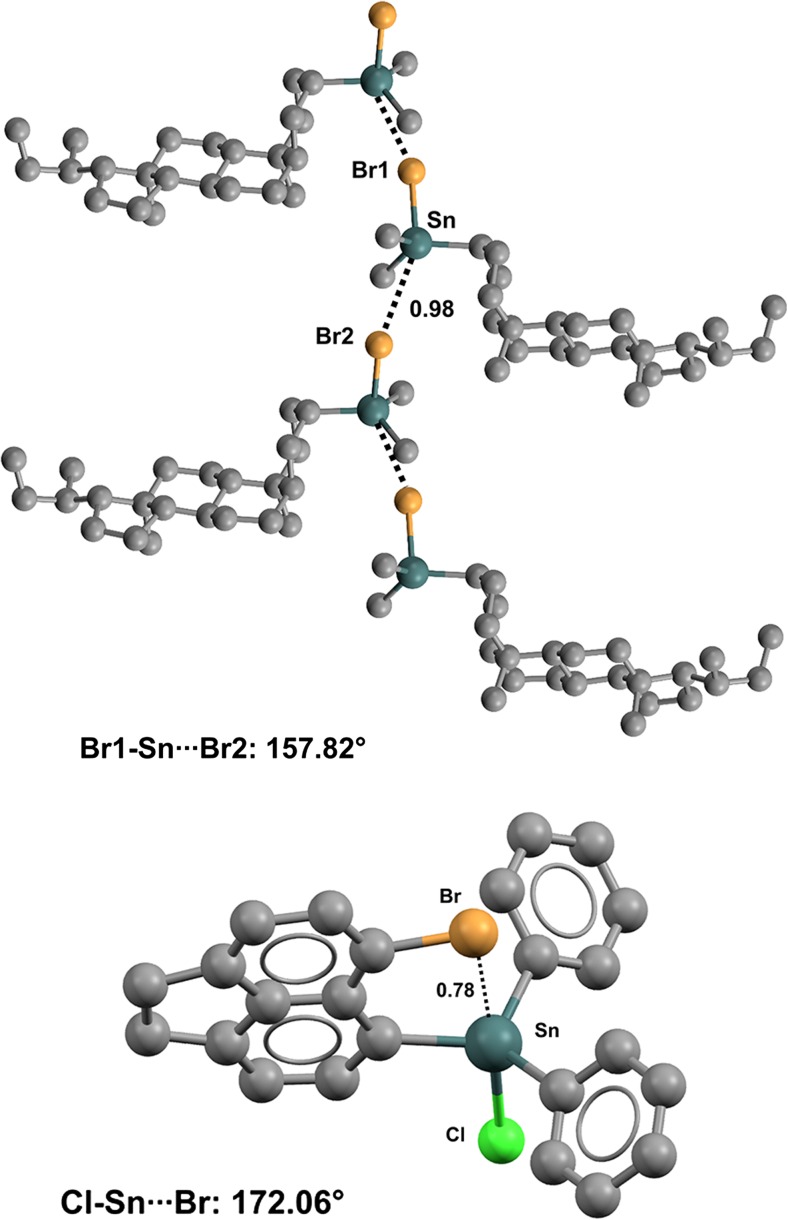
Ball and stick representations (Mercury 3.9) of the conformation adopted by chloro(6-bromo-1,2-dihydroacenaphthylen-5-yl)diphenyltin (VEKKUT,* bottom*) and the 1D chain generated by 3β-(bromodimethylstannyl)-24-nor-5β-cholane (MISYAO,* top*). TBs are depicted as* black dotted lines*; hydrogen atoms have been omitted for clarity. Nc values are shown close to the respective interactions. Color code:* gray* carbon,* brown* bromine,* dark teal* tin

The conformation of diphenyl(6-bromo-1,2-dihydroacenaphthylen-5-yl)chlorotin (refcode VEKKUT) [121] is influenced by an intramolecular TB where the bromine atom is localized along the extension of the Cl–Sn bond (Nc = 0.78; the Cl–Sn···Br angle is 172.06°) (Fig. 25, bottom) [121]. Similar Cl–Sn···Br contacts are present in various other (6-bromo-1,2-dihydroacenaphthylen-5-yl)tin derivatives. Bromine atoms can also be involved in intermolecular TBs. This is the case in the steroid derivative 3β-(bromodimethylstannyl)-24-nor-5β-cholane (refcode MISYAO) [122] (Fig. 25, top), crystals of which include infinitely long 1D chains assembled via Br–Sn···Br.

**Fig. 26:**
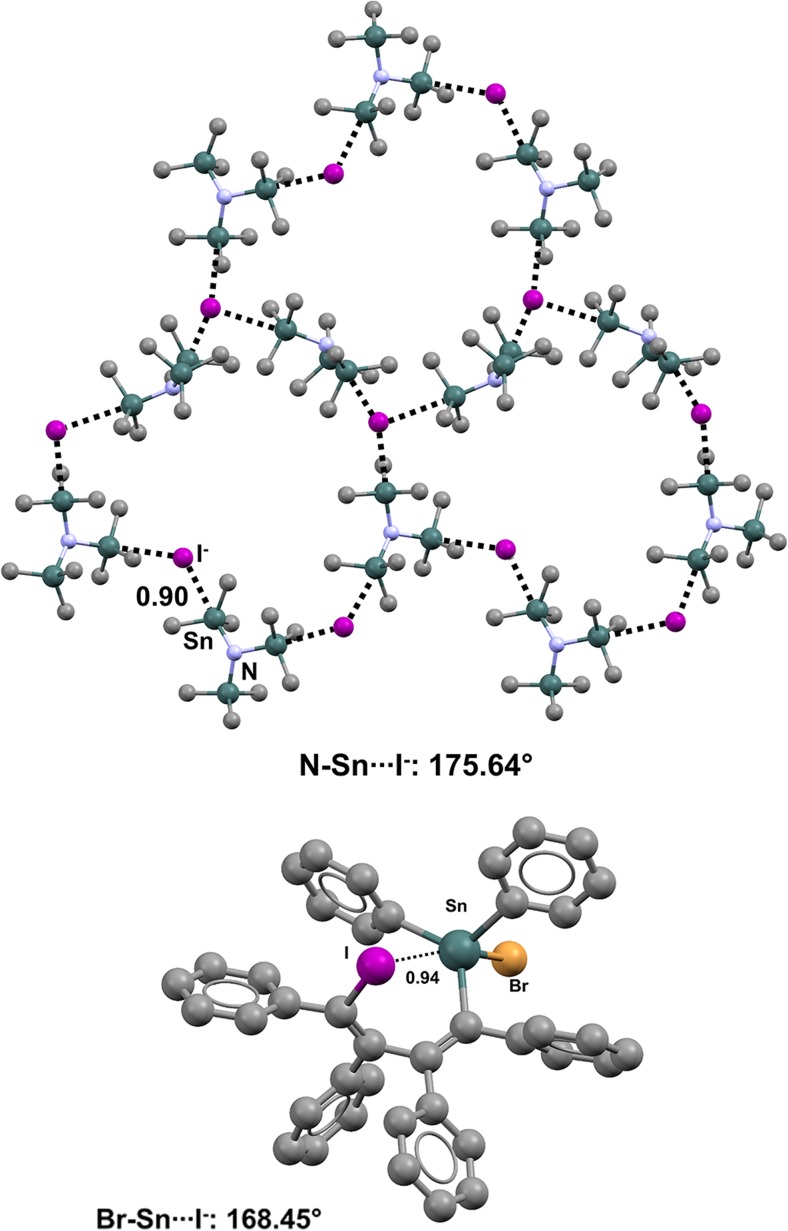
Ball and stick representations (Mercury 3.9) of the conformation adopted by bromo(4-iodo-1,2,3,4-tetraphenyl-1,3-butadienyl)diphenyltin (SICSOM,* bottom*) and the network formed by tris(trimethylstannyl)ammonium iodide (RONDAZ,* top*). One layer of RONDAZ is presented, and hydrogen atoms have been omitted for clarity. TBs are depicted as* black dotted lines*; Nc values are shown close to the respective interactions. Color code:* gray* carbon,* purple* iodine,* brown* bromine,* light blue* nitrogen,* dark teal* tin

The covalent bond pathway connecting iodine and tin in (8-iodo-1-naphthyl)trimethyltin (refcode AQIVUS) [123] is reminiscent of that connecting bromine and tin in VEKKUT, and this translates into a supramolecular similarity between the C–Sn···I TB in the former compound and the Cl–Sn···Br TB in the latter. In the crystal structure of bromo(4-iodo-1,2,3,4-tetraphenyl-1,3-butadienyl)diphenyltin (refcode SICSOM) (Fig. 26, bottom) [124], the iodine atom acts as the TB acceptor and approaches tin—the TB donor—along the extension of the Br–Sn bond (Nc = 0.94; the Br–Sn···I angle is 168.95°). This pattern is consistent with the fact that the most positive σ-hole on tin is expected to occur at this position, as bromine is more electron-withdrawing than the other atoms bound to tin. Finally, the Sn···I interactions present in crystals of tris(trimethylstannyl)ammonium iodide (refcode RONDAZ) [125] provide a nice example of charge-assisted TB. The existence of this type of TB further highlights the similarities of the different subsets of σ-hole interactions, as charge-assisted XBs [126] and charge-assisted PBs [31] have already been observed. Specifically, two crystallographically independent salt units are present in the crystal of RONDAZ; in both of these units, the tris(trimethylstannyl)ammonium cations act as tridentate TB donors and the iodide anion as a tridentate TB acceptor, and 3D networks are formed (one 3D network is shown in Fig. 26, top).

## Conclusions

In this paper, we have reported the results of an analysis of the CSD that aimed to identify crystal structures of organic derivatives of germanium and tin in which these two elements form close contacts with lone-pair-possessing atoms.

We focused our attention on close contacts where oxygen, nitrogen, and halogens were the lone-pair-possessing atoms, as a wide range of examples of those close contacts were found in the CSD. However, it may be worth mentioning that other heteroatoms (e.g., sulfur [127–129] and phosphorus [130–132]) also form similar interactions. Ether and carbonyl oxygens as well as amine, pyridine, and cyano nitrogens can all be involved in such interactions, and the geometries observed indicate that the lone pair of the heteroatom is directed towards the germanium/tin atom independent of the hybridization of the oxygen/nitrogen atom (which can be *sp*^3^, *sp*^2^, or *sp*). Close contacts are formed by derivatives in which germanium and tin atoms bear four carbon residues or where there are halogen, oxygen, sulfur, or nitrogen substituents instead of one, two, or three of those carbon residues. Regardless of the nature and hybridization state of the lone-pair-possessing atom, and independent of the nature of the residues that are covalently bound to germanium and tin, the close contacts are preferentially formed along the extensions of the covalent bonds that germanium and tin form with strongly electron-withdrawing residues. Moreover, the more electron-withdrawing the residue bound to germanium/tin, the closer the interaction along the extension of that bond.

All of these features are typical of σ-hole interactions, so we propose that the close contacts described in this review should be termed *tetrel bonds*. Tetravalent germanium and tin atoms have a tetrahedral geometry. When these atoms form one or two close contacts with lone-pair-possessing atoms, the surrounding geometry tends to change to a trigonal bipyramidal or octahedral geometry, respectively. These changes can be explained as being due to *sp*^3^ → * dsp*^3^ or *sp*^3^ →  *d*^2^*sp*^3^ rehybridization at the tetrels. They can also be rationalized by invoking the presence of a tetrel bond [133]—an attractive interaction between a lone pair and a positive σ-hole along the extension of a covalent bond formed by the tetrel. The presence of σ-holes on all four tetrels is widely supported by modeling [38–42], and is also in accord with the experimentally determined geometric features of the interactions discussed in this review. The rationale for tetrel bonding is congruent with the other alternative explanations mentioned above. However, it may offer the additional advantage that these interactions of group 14 elements can be considered to be analogous to similar interactions that occur when groups 15–18 elements function as electrophilic sites.

The examined dataset is too limited to be able to draw general conclusions, but it seems to suggest that the deviation of a tetrel bond from the extension of the relevant covalent bond to a germanium or tin atom is usually smaller than the corresponding deviations for most PBs and CBs [31]. This is consistent with theoretical calculations which show that the region of most positive electrostatic potential opposite to a covalent bond deviates from the extension of the bond to the greatest extent in pnictogen derivatives and to the least extent in tetrel derivatives [134, 135]. The greater linearity of TBs may be related to the fact that the electronic asymmetry generated around germanium and tin atoms by the four residues bonded to them is usually smaller than the electronic asymmetry generated around pnictogen and chalcogen atoms by the residues bonded to them and their lone pair(s) [136].

It also seems that steric congestion around the tetrel atoms studied in this paper plays an influential role in tetrel bond formation; such steric congestion may even prevent tetrel bond formation. For instance, tetrakis(2-fluorobenzyl)tin (refcode VULSOM) forms four intramolecular TBs, whereas its tetrakis(2-chlorobenzyl) analog (refcode CEWGEQ) forms three intramolecular TBs; also, methyltris((2-methoxymethyl)phenyl)germane (refcode IMUTEP) forms one TB, while its phenyltris((2-methoxymethyl)phenyl) analog (IMUTIT) does not form a TB.

In conclusion, the crystal structures discussed in this paper provide reliable experimental evidence that the electrophilicity of germanium and tin in some organic derivatives can be high enough that the tetrel bonds formed with a lone-pair-possessing atom help to determine the structure in crystalline solids of those derivatives. We have shown that intra- and intermolecular tetrel bonds can be found in crystals, and that these interactions can influence the preferred conformation of a molecule and/or the network of intermolecular interactions in the crystal lattice. Tetrel bonds appear to be sufficiently reliable that they could prove useful tools in crystal engineering.

## Footnotes

1 A van der Waals radius of 210 pm was adopted for germanium, as suggested by Batsanov in [47].
